# Pathogen-associated selection on innate immunity genes (TLR4, TLR7) in a neotropical rodent in landscapes differing in anthropogenic disturbance

**DOI:** 10.1038/s41437-020-0331-y

**Published:** 2020-07-02

**Authors:** Alexander Christoph Heni, Julian Schmid, Andrea Rasche, Victor Max Corman, Christian Drosten, Simone Sommer

**Affiliations:** 1 Institute of Evolutionary Ecology and Conservation Genomics, Ulm University, 89069 Ulm, Germany; 2 Smithsonian Tropical Research Institute, Balboa, Ancón Republic of Panama; 3 Institute of Virology, Charité-Universitätsmedizin Berlin, Corporate Member of Free University, Humboldt-University and Berlin Institute of Health, Berlin, Germany; 4 German Centre for Infection Research (DZIF), Berlin, Germany

**Keywords:** Immunogenetics, Ecological genetics

## Abstract

Toll-like receptors (TLRs) form part of the innate immune system and can recognize structurally conserved pathogen-associated molecular pattern (PAMP) molecules. Their functional importance in the resistance to pathogens has been documented in laboratory experimental settings and in humans. TLR diversity, however, has been rarely investigated in wildlife species. How the genetic diversity of TLRs is associated with various pathogens and how it is shaped by habitat disturbance are understudied. Therefore, we investigated the role of genetic diversity in the functionally important parts of TLR4 and TLR7 genes in resistance towards gastrointestinal nematodes and *Hepacivirus* infection. We chose a generalist study species, the rodent *Proechimys semispinosus*, because it is highly abundant in three Panamanian landscapes that differ in their degree of anthropogenic modification. We detected only two TLR7 haplotypes that differed by one synonymous single-nucleotide polymorphism (SNP) position. The TLR4 variability was higher, and we detected four TLR4 haplotypes that differed at one synonymous SNP and at three amino acid positions within the leucine-rich repeat region. Only TLR4 haplotypes had different frequencies in each landscape. Using generalized linear models, we found evidence that nematode loads and virus prevalence were influenced by both specific TLR4 haplotypes and landscape. Here, the variable “landscape” served as a surrogate for the important influential ecological factors distinguishing landscapes in our study, i.e. species diversity and host population density. Individuals carrying the common TLR4_Ht1 haplotype were less intensely infected by the most abundant strongyle nematode. Individuals carrying the rare TLR4_Ht3 haplotype were all *Hepacivirus*-positive, where those carrying the rare haplotype TLR4_Ht4 were less often infected by *Hepacivirus* than individuals with other haplotypes. Our study highlights the role of TLR diversity in pathogen resistance and the importance of considering immune genetic as well as ecological factors in order to understand the effects of anthropogenic changes on wildlife health.

## Introduction

Deforestation, agricultural encroachment, and urbanization often result in the alteration of pathogen communities and contact probability between wildlife, livestock, and humans, thereby affecting transmission and host infection patterns in multiple ways that are often intimately intertwined (Jones et al. 2008). Environmental change leading to a reduced habitat size and increasing habitat isolation can impact ecologically important host community and population traits such as species diversity and host population densities, facilitating the transmission of pathogens (Schmid et al. 2018). Habitat disturbance can also affect the individual host immune genetic constitution by increasing population isolation, and thus inhibit gene flow between subpopulations (Sommer 2005). Both ecological and genetic factors influence the prevalence and infection intensity of pathogens in wildlife reservoirs (Civitello et al. 2015; Schmid et al. 2018). However, our knowledge about the functional importance of adaptive genetic diversity in wildlife health when both pathogen loads and host genetic diversity are affected by habitat alterations and associated ecological and environmental modifications is limited.

The genetic diversity in immune-relevant genes is assumed to determine the ability of an organism to cope with changing pathogen pressure and has wide-ranging implications for population health. To date, the relationship between host genetic diversity and pathogen resistance has been mainly assessed by investigations of the major histocompatibility complex (MHC), a multigene family crucial for the adaptive immune response in vertebrates (Sommer 2005). Indeed, links between certain MHC alleles and supertypes (i.e., groups of alleles that show similar antigen-binding properties and are therefore assumed to be functionally similar) and lower parasite load and/or survival have been reported in diverse wildlife species (e.g., Schwensow et al. 2007, 2017; Froeschke and Sommer 2012). Usually populations with a large MHC allele repertoire display lower parasite loads (Meyer-Lucht and Sommer 2009).

However, host–pathogen interactions and associated immune responses are complex, and about half of the genetic plasticity towards diseases can be attributed to non-MHC genes such as cytokines and Toll-like receptors (TLRs) (Jepson et al. 1997). TLRs form part of the innate immune system, which is the first line of defense against a wide variety of pathogens, such as viruses, bacteria, and fungi (Jin and Lee 2008). They consist of a group of conserved pattern-recognition receptors that are important for recognizing pathogens. Upon activation, they trigger the production of inflammatory cytokines and co-stimulatory molecules that initiate inflammation and the activation of the adaptive immune system (Iwasaki and Medzhitov 2004; Kawai and Akira 2007; Tschirren et al. 2011). Like MHC genes, TLRs belong to a multigene family that has evolved by gene duplication (Zhou et al. 2007). Mammals usually harbor 10–12 different TLRs that are activated by distinct invariant microbial structures (Roach et al. 2005). In almost all TLRs, certain residues are under positive selection (mammals: Areal et al. 2011; apes: Wlasiuk and Nachman 2010; rodents: Tschirren et al. 2011; bats: Zhang et al. 2013; birds: Alcaide and Edwards 2011; Grueber et al. 2012; amphibians: Babik et al. 2014), and single-nucleotide polymorphisms (SNPs) are associated with disease susceptibility, especially in humans (Schröder and Schumann 2005; Wong et al. 2010; Uciechowski et al. 2011; Taylor et al. 2012; Wang et al. 2014; but see Tschirren et al. 2013).

Among the TLRs that are subject to co-evolutionary processes with pathogens are TLR4 and TLR7 (Fornůsková et al. 2013; Escalera-Zamudio et al. 2015). TLR4 detects a wide range of pathogen-associated molecular patterns (so-called PAMPs) and plays an important role in the defense against several multicellular parasites (Venugopal et al. 2008; Gavan et al. 2015) and viral diseases (Machida et al. 2006). Gastrointestinal nematodes, which are directly transmitted via free living stages, represent one of the most prevalent group of parasites (Hugot et al. 2001) and are a major cause of disease and death in humans, domestic animals, and wildlife (Stear et al. 1997). Several studies have recently emphasized the impact of historic and contemporary positive selection on the TLR4 polymorphism in rodents (Turner et al. 2012; Fornůsková et al. 2013, 2014). TLR7 belongs to the class of viral TLRs and recognizes single-stranded RNA (Heil et al. 2004). In contrast to the majority of (immune) genes (including TLR4), TLR7 is located on the X-chromosome (Fornůsková et al. 2013). The association of the individual TLR4 and TLR7 constitution and susceptibility against several diseases, such as malaria or HIV infections, has been reported in humans and mice in laboratory experimental settings (Oh et al. 2009; Skevaki et al. 2015). TLR diversity, however, has been rarely investigated in wildlife species, and our knowledge of the way in which the genetic diversity of TLRs is associated with various pathogens and is shaped by habitat disturbance remains limited (Tschirren et al. 2013; Quéméré et al. 2015).

In this study, we have taken advantage of a unique assemblage of landscapes created in the course of the Panama Canal construction and the development along the Transístmican highway. After the creation of the canal more than 100 years ago and the subsequent flooding of the area, former mountain tops have remained as protected forested islands. Large peninsulas covered with protected continuous tropical lowland forest (Barro Colorado Natural Monument) occur in the direct surroundings, and forest fragments embedded in an agricultural matrix exist about 25 km apart. Thus, a system of three different landscapes has been created with similar forest types but with different degrees of human disturbance. Recent studies have indicated that small mammal species diversity and the population density of the most abundant terrestrial mammal in the study system, namely Tome’s spiny rat *Proechimys semispinosus*, vary across the above-described landscapes (Schmid et al. 2018). Moreover, spiny rats differ in their gastrointestinal nematode richness and *Hepacivirus* prevalence because of the host environment (i.e., landscape), the associated ecological factors of species diversity and host density, and the specific MHC supertypes (Schmid et al. unpublished data). Hepaciviruses are directly transmitted from host to host. The genus *Hepacivirus* (family Flaviviridae) comprises the human hepatitis C virus (HCV), has recently been detected in a wide range of vertebrate hosts, including humans, primates, horses, cattle, bats, and rodents (Drexler et al. 2013; Corman et al. 2015), and occurs in individuals inhabiting natural and disturbed habitats (Kapoor et al. 2013; Drexler et al. 2013; Firth et al. 2014; Schmid et al. 2018).

The objective of the present study was to investigate the effect of genetic diversity encoding functionally important parts of TLR4 and TLR7, as well as ecologically important host community and population traits that are affected by anthropogenic disturbance, on the gastrointestinal nematode load and *Hepacivirus* prevalence in the widely distributed and ecologically generalistic *P. semispinosus*. We chose these two TLRs because of their above-mentioned importance in multicellular and viral pathogen resistance. Our specific aims were (1) to characterize the genetic diversity of TLR4 and TLR7 in *P. semispinosus* and to infer their phylogenetic relationship with other mammals, since TLR4 and TLR7 sequence data from wildlife species are still rare. We have also studied (2), by using generalized linear models (GLMs) (genetic models), whether TLR diversity explains gastrointestinal nematode infection intensity and *Hepacivirus* infection. We expected that nucleotide polymorphisms influence the function of TLR4 and TLR7 haplotypes in pathogen resistance. Since high MHC polymorphism is maintained by pathogen-driven selection either because of the effects of specific MHC alleles (“rare allele advantage hypothesis” or “frequency-dependent selection”, Clarke and Kirby 1966) because of an advantage of heterozygote individuals (“heterozygote advantage”, Doherty and Zinkernagel 1975), we have accordingly tested whether the “rare allele advantage hypothesis/frequency-dependent selection” or “heterozygote advantage” better explains the variance in gastrointestinal nematode loads and *Hepacivirus* resistance. Since both pathogen loads and host genetic diversity are affected by habitat alterations and associated ecological and environmental modifications, we investigated whether associations between TLR diversity and pathogen load remain stable or might be even stronger once additional landscape-specific ecological attributes (such as species diversity, host population density) are taken into account. Thus, we have investigated (3) the relative importance of TLR constitution, ecological factors, and landscape attributes in gastrointestinal nematode infection intensity and *Hepacivirus* infection by comparing the best genetic model with the best ecology & landscape model (as determined by Schmid et al. 2018) and with an integrated genetic & landscape model. Our study emphasizes the value of embedding studies of the functional importance of immune genetic diversity in wildlife health into a landscape context, since both pathogen loads and TLR diversity are affected by habitat alterations and associated ecological and environmental modifications.

## Materials and methods

### Study area and sampling

**Fig. 1:**
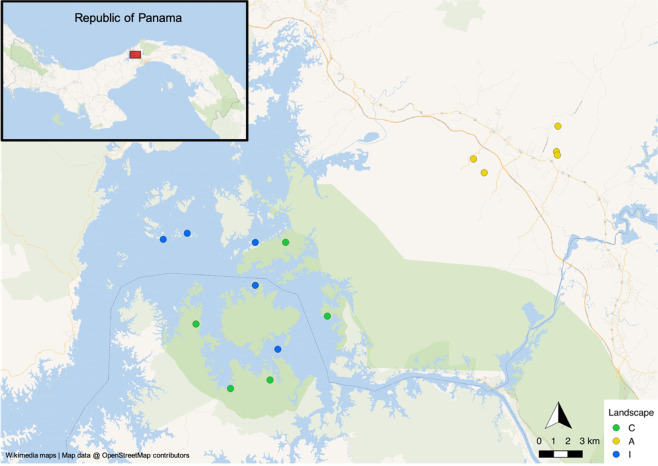
Geographical location of the study sites in Central Panama. Study sites in continuous forests (landscape C) on large peninsulas around Gatun Lake are labeled in green, study sites on forested islands (landscape I) surrounded by water are marked in blue, and forest fragments in an agricultural matrix situated in the vicinity of human settlements and roads (landscape A) are in yellow. The distance between the study sites of landscapes C + I and A is approximately 25 km. The current terrestrial fauna of the islands is probably descended from individuals inhabiting the area before isolation. Occasional re-colonization by animals floating on plant material from neighboring islands or the mainland might also have occurred. The figure was created with QGIS 2.18 (QGIS Development Team 2016).

The study was conducted in the Panama Canal area, Central Panama, during two sessions of fieldwork (season 1: October 2013–May 2014, season 2: October 2014–May 2015) (Fig. 1). Live-trapping of small mammals took place in three landscapes differing in their extent of anthropogenic environmental change. Five independent study sites were investigated in each landscape (in total 15 study sites, Supplementary Table S1, see Schmid et al. 2018 for details). Study sites within the continuous lowland tropical forest (landscape C) surrounding Gatun Lake served as a control with little human impact. The second landscape (=agricultural landscape, landscape A) consisted of smaller forest fragments (1.5–51 ha) embedded in an agricultural matrix. The third landscape comprised forested islands (5.2–17.5 ha, landscape I) located inside the Canal Zone and was formed approximately 100 years ago during the construction of the Panama Canal and its subsequent flooding (Fig. 1).

All study sites are separated by water, streets, or roads. Capture/re-capture and radio-telemetry studies estimated the mean home-range size as between 178 and 2375 m^2^, with a core home range between 39 and 469 m^2^ (Adler et al. 1997; Seamon and Adler 1999; Endries and Adler 2005). Thus, the population of each study site can be considered as independent from those of other study sites. At each of the 15 study sites, 100 trap stations separated by a distance of 20 m were established along four transects. Each trapping station consisted of one Tomahawk trap (15.2 cm × 15.2 cm × 48.3 cm, http://www.livetrap.com) and two Sherman traps (10.2 cm × 11.4 cm × 38.1 cm, http://www.shermantraps.com), i.e., 300 traps were set up in total. Traps were opened at dusk and baited with a mixture of peanut butter, banana, bird seeds, dog food, and oat flakes for five nights, and were examined and closed the following morning at dawn. Ecological data and samples for genetic analyses were collected as described previously (Schmid et al. 2018), and animals were released after being handled at the respective trapping location. We determined the age (adult or juvenile) based on pelage characteristics (Adler 1994). For the present study, we investigated a subset of previously studied individuals (Schmid et al. 2018), aiming at a balanced data set across landscapes (Supplementary Table S1). The project was ethically approved (Smithsonian IACUC protocol 2013-0401-2016-A1-A7), and the fieldwork was conducted with the permission of the Panamanian Government, which also gave permission for the exportation of the collected samples to Germany (SE/A-21-14, SE/A-69-14, and SEX/A-22-15). Details of the sampled individuals (i.e. sampling location, TLR haplotypes, infection status, sex, and age) are provided in Supplementary File 1.

### Detection of the virus and macroparasite infection

Blood samples were screened for *Hepacivirus* infections, as Hepacivirus is known to be a common pathogen in a wide range of rodents and other mammals (Drexler et al. 2013; Corman et al. 2015; Schmid et al. 2018). Details of the laboratory protocols are described in Schmid et al. 2018 and in Supplementary File 2.

We further examined the prevalence and infection intensity of gastrointestinal nematodes. Individual fecal egg counts (FEC) were calculated by using a modification of the widely used McMaster technique (Sloss et al. 1994; used in Schad et al. 2005; Meyer-Lucht and Sommer 2005; Axtner and Sommer 2007; Schwensow et al. 2007; Froeschke and Sommer 2012). We used this standardized approach to count helminth eggs by diluting them in iodine–potassium solution with a specific density of 1.5 g/mL, thus enhancing the detectability of eggs with high specific weights, such as nematode eggs (Thienpont et al. 1986). McMaster slides were used to inspect the samples for the presence of various eggs by means of an optical microscope. All eggs were measured using a calibrated eyepiece graticule, which allowed their grouping into egg morphotypes and to count their abundance. This approach has been intensively tested in Froeschke et al. (2010), where both FEC and dissected individuals were investigated and all abundant egg morphotypes could be linked to adult worms found in the gastrointestinal tract. Photographs are available on request from the author. Prior to subsequent analyses, we tested the effect of field season on nematode infection intensity by using the Wilcoxon rank-sum test for independent samples with continuity correction and for *Hepacivirus* virus prevalence by applying the Pearson's chi-squared test with Yates' continuity correction. For both pathogen taxa, no significant effect of the field season was detected (nematodes: *W* = 9224.5, *p* value = 0.06; *Hepacivirus*: *χ*^2^ = 2.406, d.f. = 1, *p* value = 0.12); nevertheless, we also present models with “season” included.

### Amplification and sequencing of TLR genes

DNA was extracted from skin tissue by using the NucleoSpin® Tissue Kit from Machery-Nagel, following the instructions of the manufacturer. Primers were constructed to target the functionally important leucine-rich repeat (LRR) domain of the TLR4 and TLR7 genes, identified by using the online tool LRRfinder (www.lrrfinder.com) in the closely related degu (*Octodon degus*). A similar location was assumed in the study species. For primer design, TLR4 and TLR7 sequences of several rodent species (see Supplementary Table S2) closely related to our study species were aligned. Primers were developed targeting conserved sites between the mentioned species by using Primer 3 (Koressaar and Remm 2007) implemented in Geneious Version 8 (http://www.geneious.com, Kearse et al. 2012). The TLR4-primer pair (forward primer: 5′-ACCTGACCAACTTGGAGTACT-3′, reverse primer: 5′-CCAACAGAGGTGCGAACAGT-3′) amplified a 574-base-pair (bp) region, and the TLR7 primers targeted a region of length 998 bp (TLR7 forward primer: 5′-ACTGGTCAACATAGAAATGCT-3′, reverse primer: 5′-ACCTTGGACCTGAGTAGAAA-3′).

The designated region of TLR4 was amplified in a volume of 10 µL, consisting of 5 µL of DreamTaq^TM^ PCR Master Mix (Thermo Scientific^TM^), 0.2 µL of each primer (10 µM), 3.6 µL of demineralized water, and 1 µL of template DNA. PCRs for TLR7 were set at a volume of 15 µL, with 7.5 µL of DreamTaq^TM^ PCR Master Mix (Thermo Scientific^TM^), 0.3 µL of each primer (10 µM), 5.4 µL of demineralized water, and 1.5 µL of template DNA.

The PCR protocol for TLR4 consisted of an initial denaturation step of 90 s at 95 °C, followed by 30 cycles of 30 s at 95 °C, 30 s at 55 °C, and 60 s at 72 °C, followed by a final elongation of 10 min at 72 °C. A similar protocol was used for TLR7, but with 40 cycles and an annealing temperature of 52 °C. PCR products were cleaned by using FastAP/Exo nucleases (Thermo Scientific^TM^) and Sanger-sequenced on an ABI 3130 Genetic Analyzer (Applied Biosystems^TM^) with an injection time of 12 s for TLR4 and 30 s for TLR7. For the separation of the genotype into haplotypes, i.e., to allow consideration of the nucleotide sequence of one chromosome only, the PHASE algorithm (Stephens et al. 2001) implemented in DnaSP Version 5 (Librado and Rozas 2009) was applied. This was an easy and straightforward approach since we did not observe any evidence for copy number variation nor length variation in the Sanger sequences.

We started the project by analyzing TLR7, but since the observed diversity and d*N*/d*S* ratio was low, and because the results did not change with increasing sample size (see below), we concluded that a sample size close to 100 individuals (*N* = 95) was sufficient to address our research questions. We thus additionally focused on TLR4 and genotyped *N* = 158 individuals, aiming at a minimum sample size of 10 individuals per study site (Supplementary Table S1).

### Data analysis and statistics

The genetic diversity of TLR4 and TLR7 in *P. semispinosus* was characterized by using Geneious Version 8. The haplotype and genotype frequencies and the number of heterozygotes were inferred in the overall data and per landscape (with five independent study sites in each landscape) by using Arlequin ver. 3.5.2.2 (Excoffier and Lischer 2010). Arlequin was also used to test for differences in TLR haplotype frequencies between landscapes by means of the global test of differentiation (100,000 Markov steps). Further, for each individual haplotype, chi-square tests were performed by using R ver. 3.6.3 (R Core Team 2020) to test whether frequencies differed between the landscapes.

The phylogenetic relationships among the available TLR gene sequences of diverse mammalian, monotremata, marsupial, and bird species (see Supplementary Table S2) were investigated after identification of the best model by using the model selection implemented in MEGA X (Kumar et al. 2018). TLR4 and TLR7 sequences of a reptile (green sea turtle *Chelonia mydas*) were used as the outgroup to root the tree. The phylogenetic tree for TLR4 (597 bp long) was constructed by using maximum likelihood and the general time-reversible model with gamma distribution with invariant sites. The TLR7 tree (700 bp long) was constructed by using the maximum likelihood and the Kimura 2-parameter model with gamma distribution with invariant sites. The phylogenetic trees were built with 10,000 bootstrap replicates that were subsequently condensed by using a 50% cut-off value.

We investigated the influence of the TLR4 and TLR7 constitution on nematode infection intensity and *Hepacivirus* prevalence by using GLMs in the library MASS in R ver. 3.6.3 (R Core Team 2020). This approach was also used in a previous investigation aimed at understanding the effects of ecological factors associated with pathogen prevalence (Schmid et al. 2018) and thus allowed a direct comparison of the results.

The association between the individual TLR haplotypes and nematode infection intensity was tested by using a GLM with a negative binomial error structure constructed with the MASS package (Venables and Ripley 2002), because of the strong overdispersion of FEC. A binomial error structure was applied for the *Hepacivirus* prevalence. We tested whether “rare allele advantage hypothesis/frequency-dependent selection” or “heterozygote advantage” better explained the variance. For this purpose, we constructed genetic models with either one of the haplotypes or heterozygosity (i.e., “heterozygous genotype”) as the explanatory factor. Models for TLR4 and TLR7 were constructed separately to minimize the number of confounding factors.

Since both pathogen loads and host genetic diversity are affected by habitat alterations and associated ecological and environmental modifications, we further investigated whether the detected genetic associations between TLR diversity and pathogen load explain the variance in pathogen loads equally well as ecological parameters or even better if landscape-specific ecological attributes are taken into account. Therefore, we compared the AICc (Akaike information criterion with a correction for small sample size) of the genetic models with the AICc of the ecology & landscape model (as determined by Schmid et al. 2018), which tested host population density in interaction with sex ratio, sex, host age, and landscape as explanatory variables for the pathogen load, and with a genetic & landscape model (this study). We only included one interaction (host density:sex ratio) because it was determined as an important contributor by Schmid et al. (2018).

The genetic & landscape model included the parameters of the best genetic model plus landscape. The term “landscape” served here as a surrogate, reflecting that the most important and influential ecological factors distinguishing landscapes were species diversity and host population density (see Schmid et al. 2018).

The model with the lowest AICc was termed the best whereby all models with a delta AICc <2 were considered as equal in explaining the differences in infection. The summary of the best models explaining nematode infection intensity and *Hepacivirus* prevalence was called. Post hoc tests (Tukey method) for the best models were obtained using emmeans (Lenth 2020) and the effect size (odds ratio) and confidence intervals for the significant TLR-factors were calculated by using *sjPlot* (Lüdecke 2020). All analyses followed the recommendations of Zuur et al. (2010), and the corresponding graphs were obtained by using the R-packages *ggplot2* (Wickham 2009) and *cowplot* (Wilke 2017).

## Results

### TLR diversity and phylogenetic relationships

In the highly abundant studied rodent, four TLR4 haplotypes differing in their amino acid sequence (Genbank accession numbers MT136926–MT136929, Supplementary Table S3) were detected in 158 individuals (C:N = 53, A:N = 52, I:N = 53, Supplementary Table S3). The haplotypes were based on three polymorphic amino acid positions within the LRR region (Arg185Trp, Ala234Val/Glu, and Ile331Met) and one synonymous SNP (Leu184Leu; numbers refer to the position in the amino acid sequence of *O. degus* (XM_004641503.2); d*N*/d*S* ratio = 3.0).

Whereas TLR4_Ht1 and TLR4_Ht2 were similarly abundant (present in 70.9% and 76.0% of all individuals, respectively), TLR4_Ht3 (5.1%) and TLR_Ht4 (9.6%) were less often encountered. All possible genotype combinations between the four haplotypes were retrieved. The rarest genotype occurred in one individual (TLR4_Ht3.TLR4_Ht4 and TLR4_Ht4.TLR4_Ht4) and the most common genotype in 78 individuals (TLR4_Ht1.TLR4_Ht2). In total, 61 animals (38.6%) were homozygous for one genotype or the other, and 97 individuals (61.4%) were heterozygous.

Two different TLR7 haplotypes were detected in 95 individuals (C:N = 46, A:N = 9, I:N = 40, Supplementary Table S3). These two haplotypes (Genbank accession numbers MT136930–MT136931) differed by one synonymous mutation at nucleotide position 1148 (nucleotide C → T, Asn382Asn, number refers to the amino acid sequence of *O. degus* (XM_004641503.2), d*N*/d*S* ratio = 0).

TLR7_Ht1 was encountered in 62.1%, and TLR7_Ht2 in 69.5% of the individuals. Because of the location of the TLR7 gene on the X-chromosome, five different genotypes were retrieved: homozygous for TLR7_Ht1 with one copy (males only, 20 animals), homozygous for TLR7_Ht1 with two copies (females only, 9 animals), heterozygous (females only, 30 animals), homozygous for TLR7_Ht2 with one copy (males only, 15 animals), and homozygous with two copies (females only, 21 animals).

The phylogenetic relationship of the TLR4 sequences of *P. semispinosus* and of a further 50 taxa revealed two main branches, one including all the mammal species and one including the birds, with the reptiles as an outgroup (Supplementary Fig. 1). Many orders were clearly resolved (e.g., Chiroptera), whereas others were dispersed across several lineages (e.g., rodents).

The results for TLR7 (Supplementary Fig. 2) were similar, with one main branch for the mammals (all taxa expected the Aves clade and the *Chelonia mydas*) and one for the Aves, with the reptiles as an outgroup. In both phylogenetic trees, the spiny rat was placed next to its closest relative, the *O. degus*.

### Differences in pathogen load and TLR haplotype frequencies across landscapes

**Fig. 2:**
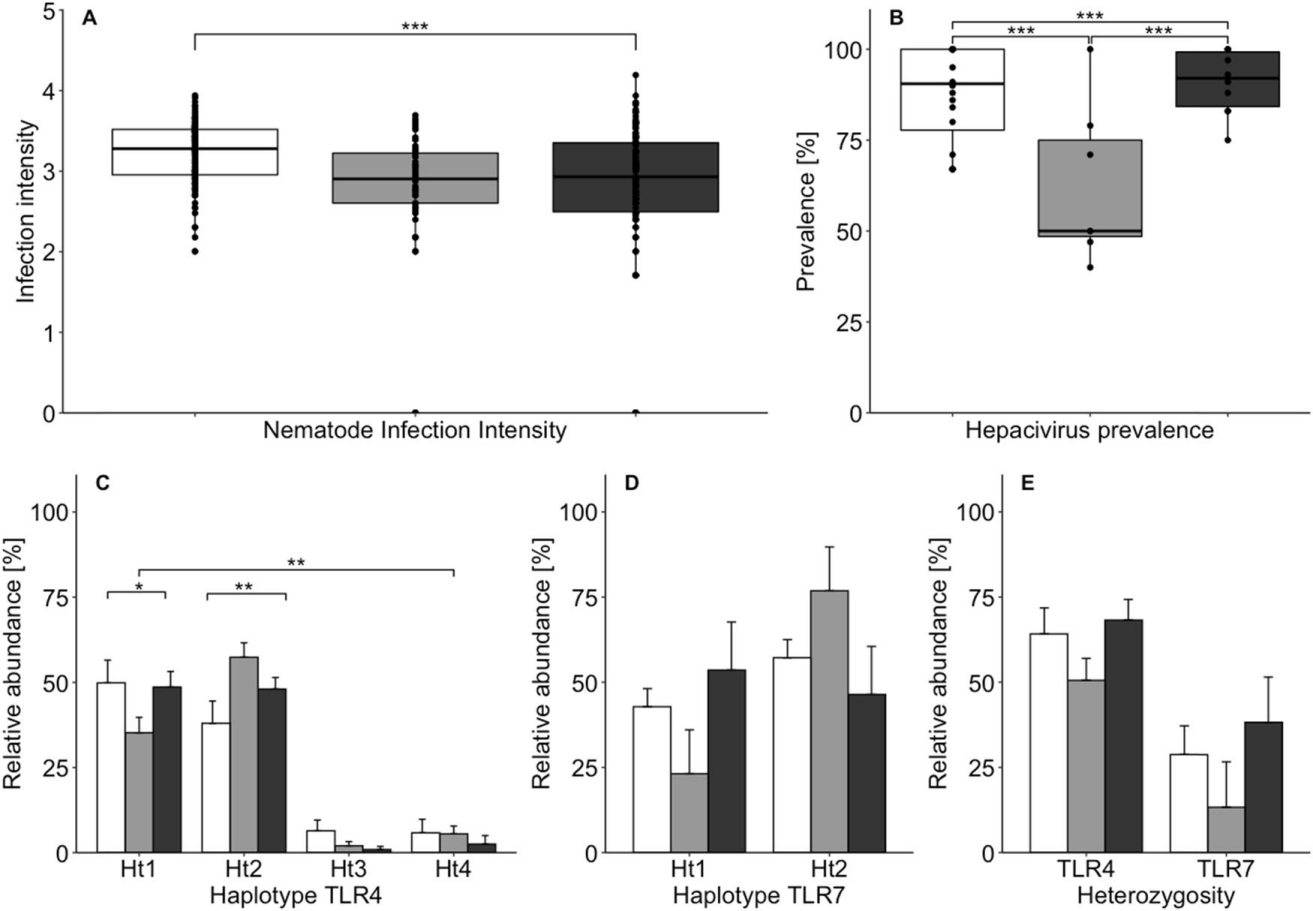
Pathogen loads and TLR haplotype frequencies of spiny rats across landscapes differing in anthropogenic disturbance. **a** Infection intensity (log10 (FEC + 1)) of the most common nematode (this study, *n* = 293) and **b** *Hepacivirus* prevalence (data taken from Schmid et al. 2018, *n* = 673) in *P. semispinosus*. The boxplots indicate the median, 25 and 75% quartiles, and minimum and maximum values. Relative abundance of **c** TLR4 haplotypes (*n* = 158), **d** TLR7 haplotypes (*n* = 95), and **e** heterozygosity of TLR4 (*n* = 158) and TLR7 (*n* = 95) across the three landscapes C, A, and I in central Panama. The bars indicate the mean relative abundance of the five study sites per landscape±the standard error. Boxplots and bars representing the continuous forest (landscape C) are labeled in white, forested fragments embedded in an agricultural landscape (landscape A) in light gray, and forested islands in the Gatun lake (landscape I) in dark gray. *p < 0.05, **p < 0.01, ***p < 0.001.

In *P. semispinosus*, we distinguished 14 different nematode morphotypes (Supplementary File 3). The most common nematode (nematode morphotype 3, overall prevalence: 96.3%) was present in all three landscapes (C = 100%, A = 97.1%, I = 92.7%, Supplementary Fig. 3), with an FEC ranging from 25 to 7800 per gram feces. Four nematodes (nematode morphotypes 1, 6, 7, and 11) were detected with a moderate prevalence (14.0–37.8% of the individuals, but unevenly distributed across the landscapes limiting statistical investigations), whereas the nine remaining nematode morphotypes occurred at a low frequency (overall prevalence: 0.3–6.5%) (Supplementary Fig. 3). In order to be able to disentangle the genetic and environmental effects on infection intensity, we restricted our analyses to the most common nematode (nematode morphotype 3) present in a large number of individuals in all three landscapes. The infection intensity with the most common nematode was higher in individuals inhabiting landscape C compared with rats living in landscapes A and I (Kruskal–Wallis rank-sum test: *χ*^2^ = 27.287, d.f. = 2, *p* < 0.001, Fig. 2a). Dunn's test for post hoc comparisons showed a difference between C and A (*Z* = 4.57, *p* adj. = 1.45e−05) and C and I (*Z* = 4.39, *p* adj. = 1.72e−05), but not for A and I (*Z* = −0.81, *p* adj. = 0.42, Fig. 2a).

As reported by Schmid et al. (2018), *Hepacivirus* prevalence was significantly lower in the study sites of landscape A than in landscape C and landscape I, but did not significantly differ between landscapes C and I (GLM, Gaussian error structure: A–C *t* = 4.05, *p* < 0.001; A–I *t* = 4.09, *p* < 0.001; C–I *t* = 0.61, *p* = 0.55, Fig. 2b, *Hepacivirus* data taken and figure modified from Schmid et al. 2018).

All four TLR4 haplotypes were detected in all three landscapes (C, A, and I), but their frequencies differed (global test of differentiation: exact *p* value = 0.01 + −0.003). TLR4_Ht1 was less abundant in landscape A than in landscapes C and I (*χ*² = 8.10, d.f. = 2, *p* = 0.02), whereas TLR4_Ht2 showed the opposite pattern and was more abundant in A than in the two other landscapes (*χ*² = 9.23, d.f. = 2, *p* = 0.01) (Fig. 2c). Differences in the haplotype frequency of TLR4_Ht3 and TLR4_Ht4 could not be tested because of their overall low presence.

Both TLR7 haplotypes were encountered in all three landscapes with no sign of population differentiation based on haplotype frequencies (global test of differentiation: exact *p* value = 0.75 + −0.01): TLR7_Ht1 was slightly less frequent in A (0.36) than in C (0.42) and I (0.47), whereas TLR7_Ht2 showed the corresponding opposite pattern (Fig. 2d).

Calculation of HWE revealed a significant excess of TLR4-heterozygote animals in the landscape C (*H*_obs_ = 0.642, *H*_exp_ = 0.608, *p* = 0.007). Further, more heterozygote than homozygote animals were observed than expected in landscape I, but this deviation was not significant (*H*_obs_ = 0.679, *H*_exp_ = 0.541, *p* = 0.127). Homozygote and heterozygote individuals were equally abundant in landscape A (*H*_obs_ = 0.519, *H*_exp_ = 0.544, *p* = 0.478) (Fig. 2e).

Concerning TLR7, HWE tests showed no deviation between the number of observed and expected heterozygous individuals in any landscape (landscape C: *H*_obs_ = 0.320, *H*_exp_ = 0.470, *p* = 0.186; landscape A: *H*_obs_ = 0.400, *H*_exp_ = 0.533, *p* = 1.000; landscape I: *H*_obs_ = 0.600, *H*_exp_ = 0.497, *p* = 0.414) (Fig. 2e).

### Effects of TLR4 diversity, ecological factors, and landscape on nematode infection intensity

**Fig. 3:**
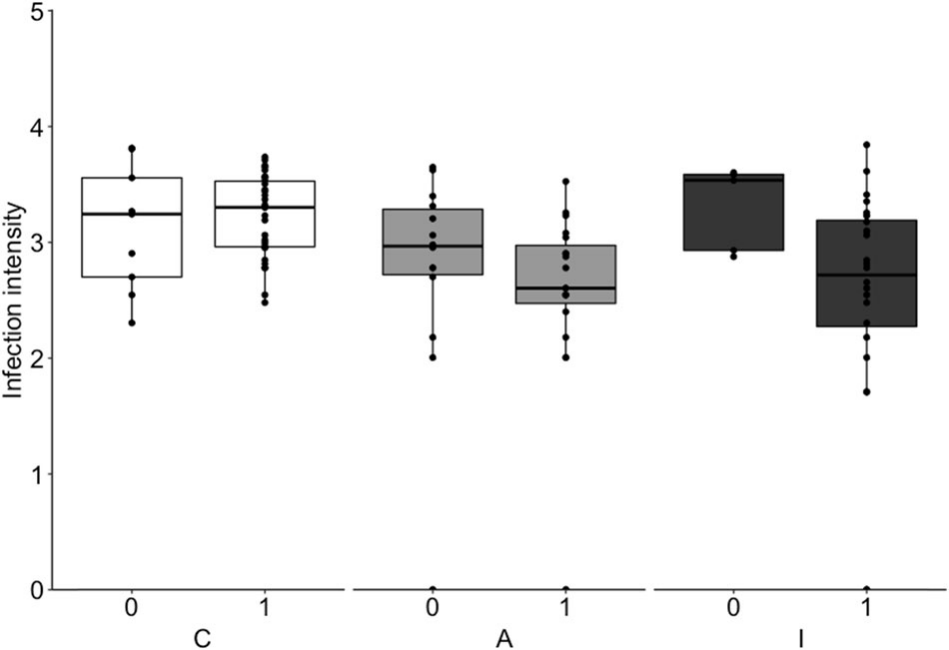
Effect of carrying TLR4_Ht1 (absence (0), presence (1)) on the infection intensity (log10 (FEC + 1)) of the most common nematode across landscapes (*N* = 105, Supplementary Table S1). The boxplots indicate the median, 25 and 75% quartiles, and minimum and maximum values. Boxplots representing continuous forest (landscape C) are labeled in white, forested fragments embedded in an agricultural landscape (landscape A) in light gray, and forested islands in the Gatun lake (landscape I) in dark gray.

All genetic models including TLR heterozygosity or one of the four haplotypes explained the variance in nematode infection intensity equally well (delta AICc < 2, Table 1). Their AICc values were comparable to those of the ecology & landscape model consisting of landscape, sex, age, host density in interaction with sex ratio as explanatory factors (delta AICc < 2). However, the model combining the genetic factors and landscape (genetics & landscape model) resulted in the lowest AICc of all the models tested (Table 1). The model analyses revealed that the variance in nematode infection intensity was best explained by TLR4_Ht1 and the landscape with a delta AICc >2 compared to all other genetics & landscape models. The post hoc test for the best model (Table 2, Supplementary Table S4) confirmed that both the landscape and TLR4_Ht1 have a significant impact on nematode infection intensity (Fig. 3). The presence of TLR4_Ht1 is associated with a lower nematode infection intensity (effect size for TLR4_Ht1: odds ratio = 0.61, 95% confidence intervals: lower = 0.36 upper = 0.99).

**Table 1:** Comparison of models explaining the association between TLR4 constitution, ecological & landscape factors, and nematode infection intensity (*n* = 105).

Models	d.f.	AICc	delta
*1. Genetic models*			
Heterozygosity	3	1757.436	8.563
TLR4_Ht1	3	1756.212	7.339
TLR4_Ht2	3	1757.515	8.642
TLR4_Ht3	3	1755.735	6.862
TLR4_Ht4	3	1757.519	8.646
*2. Ecological & landscape model*			
Landscape, sex, age, density:sex ratio	7	1756.384	7.511
*3. Genetics & landscape models*			
Heterozygosity, landscape	5	1752.337	3.464
**TLR4_Ht1, landscape**	**5**	**1748.873**	**0**
TLR4_Ht2, landscape	5	1752.868	3.995
TLR4_Ht3, landscape	5	1751.025	2.152
TLR4_Ht4, landscape	5	1752.697	3.824

Models were split into three different categories: (1) genetic models, investigating the effects of different TLR haplotypes and heterozygosity; (2) ecological & landscape model, investigating the landscape, sex, age, and host population density in interaction with sex ratio as factors; and (3) genetics & landscape models, combining genetic factors from the genetic models with landscape as a surrogate for ecological factors. The best model is shown in bold.

**Table 2:** Post hoc test for factors of the best model (genetic & landscape model) explaining the variance in infection intensity with the most common nematode.

Contrast	Ratio	SE	d.f.	*z* ratio	*P*
C–A	2.556	0.683	Inf	3.513	<0.001
C–I	1.745	0.46	Inf	2.115	0.087
A–I	0.683	0.192	Inf	−1.353	0.366
TLR4_Ht1 (absent–present)	1.65	0.418	Inf	1.978	0.048

Note that the post hoc test for landscape and TLR4_Ht1 were run separately and the results are back transformed from log scale (*N* = 105).

### Effects of TLR4 diversity, ecological factors, and landscape on *Hepacivirus* prevalence

**Fig. 4:**
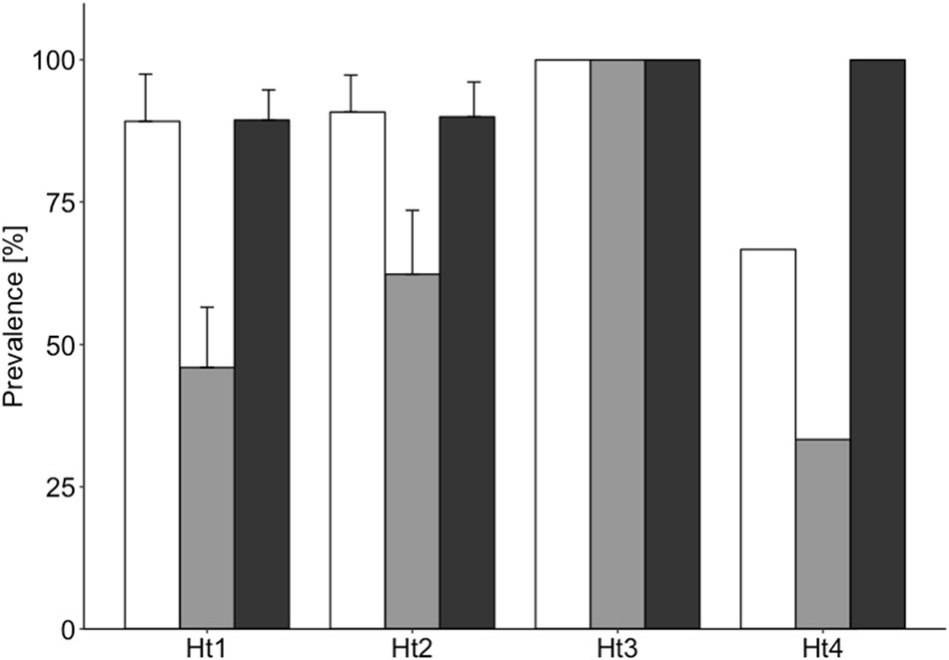
Effect of carrying TLR4_Ht1 to TLR4_Ht4 on *Hepacivirus* prevalence across landscapes (*N* = 149). The bars indicate the mean relative abundance of the haplotype in infected individuals per landscape ± the standard error for the two most common haplotypes (error bars could not be calculated for TLR4_Ht3 and TLR4_Ht4 because of their rarity in our study). Bars representing continuous forest (landscape C) are labeled in white, forested fragments embedded in an agricultural landscape (landscape A) in light gray, and forested islands in the Gatun lake (landscape I) in dark gray.

With regard to TLR4 and *Hepacivirus* infection, the genetic models including TLR4_Ht3 or TLR4_Ht4 explained the variance in prevalence equally well (with a delta between these two models of <0.1) (Table 3). The ecology & landscape model had a considerably lower AICc, and the AICc decreased even further by combining a genetic factor (either TLR4_Ht3 or TLR4_Ht4) and landscape (genetics & landscape model). The post hoc tests for the best models, however, indicated that landscapes differed significantly regarding the *Hepacivirus* infection status, while the haplotype TLR4_Ht3 had no effect (Table 4a, Supplementary Table S5a) and the haplotype TLR4_Ht4 had only a marginal effect (*p* = 0.068; Table 4b, Supplementary Table S5b) (Fig. 4).

**Table 3:** Comparison of models explaining the association between TLR4 constitution, ecological & landscape factors and *Hepacivirus* infection status (*n* = 149).

Models	d.f.	AICc	Delta
*1. Genetic models*			
Heterozygosity	2	166.415	31.467
TLR4_Ht1	2	166.174	31.226
TLR4_Ht2	2	166.434	31.486
TLR4_Ht3	2	162.109	27.161
TLR4_Ht4	2	162.178	27.230
*2. Ecological & landscape model*			
Landscape, sex, age, density:sex ratio	7	136.798	1.850
*3. Genetics & landscape models*			
**TLR4_Ht3, landscape**	**4**	**134.948**	**0.000**
**TLR4_Ht4, landscape**	**4**	**135.717**	**0.769**

Model were split into three different categories: (1) genetic models, investigating the effects of different TLR haplotypes and heterozygosity; (2) ecological & landscape model, investigating landscape, sex, age, and host population density in interaction with sex ratio as factors (Schmid et al. 2018); and (3) genetics & landscape models, combining genetic factors from the genetic models with landscape as a surrogate for ecological factors. The best model is shown in bold.

**Table 4:** Post hoc test for factors of the best model (a) TLR4_Ht3 and landscape and (b) TLR4_Ht4 and landscape from the Genetics & landscape model) explaining the variance in *Hepacivirus* prevalence.

Contrast	Ratio	SE	d.f.	*z* ratio	*P*
*(a) TLR4_Ht3 and landscape*					
C–A	2.374	0.599	Inf	3.966	<0.001
C– I	0.054	0.706	Inf	0.077	0.997
A–I	−2.320	0.552	Inf	−4.206	<0.001
TLR4_Ht3 (absent–present)	−16.3	1243	Inf	−0.013	0.990
*(b) TLR4_Ht4 and landscape*					
C–A	2.458	0.606	Inf	4.058	<0.001
C–I	0.243	0.713	Inf	0.341	0.938
A–I	−2.215	0.554	Inf	−3.999	<0.001
TLR4_Ht4 (absent–present)	1.21	0.661	Inf	1.827	0.068

Note that the post hoc test for landscape and TLR4_Ht1 were run separately (*N* = 149).

### Effects of TLR7 diversity, ecological factors, and landscape on *Hepacivirus* prevalence

The models constructed to understand the association between TLR7 diversity and *Hepacivirus* prevalence showed that all genetic models had similar AICc with a delta <0.32 (Supplementary Table S6). The genetic models explained the variance slightly better than the ecology & landscape model and the genetics & landscape model, but all models revealed a delta AICc <2 (Supplementary Table S6). The post hoc test for the best model, however, showed that the absence or presence of TLR7_Ht1 did not influence the *Hepacivirus* infection status (Supplementary Tables S7 and S8). The *Hepacivirus* prevalence in individuals carrying TLR7_Ht1 or TLR7_Ht2 was 84.6% and 86.9%, respectively. Including “season” in the ecology & landscape models did not affect the results (all delta AICc < 2, Supplementary Tables S9–S11).

## Discussion

Immune genes represent the most rapidly evolving parts of the genome, because of the diverse and constant selection pressure from rapidly evolving pathogens (Sommer 2005; Piertney and Oliver 2006). Studies on TLRs' molecular evolutionary dynamics have revealed, however, that selective pressures vary between the TLRs and taxa, and, to date, the role of TLR polymorphism and the type of selection that shapes this polymorphism in natural host populations is poorly understood (Grueber et al. 2012; Tschirren et al. 2013; Babik et al. 2014), particularly when compared with their adaptive counterparts, namely the MHC receptors, of the adaptive branch of the immune system. Moreover, most studies concerning the role of immune diversity in TLRs to date have focused either on model species (e.g., Palermo et al. 2009; but see Tschirren et al. 2012) or on critically endangered or threatened species (e.g., Grueber et al. 2012, 2015; Cui et al. 2015a, b). Few studies to date have investigated the genetic diversity of TLRs across landscapes (e.g., Quéméré et al. 2015) or its role in association with pathogens (Tschirren et al. 2013), although its importance has been depicted in association with fitness, resistance to diseases, and survival in wildlife (Cui et al. 2015a, b). The purpose of our study was to investigate the diversity of TLR4 and TLR7 in a widely distributed rodent and its role in resistance to gastrointestinal nematode burden and *Hepacivirus* infections. Thereby, we were specifically interested in whether TLR diversity by means of specific haplotypes or genotype heterozygosity explains gastrointestinal nematode infection intensity and *Hepacivirus* infection, and whether this association is impacted by environmental changes affecting host ecology. Thus, we aimed at understanding the relative importance of TLR constitution, ecological factors, and landscape attributes on the infection risk.

### Diversity and phylogenetic relationship of TLR4 and 7 haplotypes detected in *P. semispinosus*

Both TLR4 and TLR7 of Tome’s spiny rat *P. semispinosus* were located in phylogenetic trees within a branch of representatives of the suborder *Hystricomorpha* next to its closest relative, the degu (*O. degus*) (Lacher et al. 2016). Since TLRs are ancient receptors and are important for maintaining the immune system, the placement according to phylogeny is as expected (Roach et al. 2005). In *P. semispinosus*, the genetic diversity was higher in TLR4 (four haplotypes, one synonymous, and three non-synonymous nucleotide exchanges, *N* = 158 individuals) than in TLR7 (two haplotypes, one synonymous position, *N* = 95 individuals). In both receptor-encoding genes, the encountered polymorphisms were located within the LRR region and, thus, might have direct effects on binding probabilities with pathogens. Although silent mutations are traditionally assumed to have no impact on the binding probabilities, they can still have an impact on the functionality, such as pathogen resistance (e.g. Wada et al. 2009; Kim et al. 2010; Brest et al. 2011; Sauna and Kimchi-Sarfaty 2011). The mechanisms are still not fully resolved, but linkage to other areas of the genome and, thus, impacts on, for example, promoters or signaling cascades within other areas of the receptor have been suggested.

A higher genetic diversity for TLR4 than for TLR7 has also been detected in a recent study across 23 rodent species (Fornůsková et al. 2013), and similar results have been shown by Liu et al. (2017) in wolves. Variation in TLR diversity is most likely a result of variable pathogen pressures. Several studies have found a high degree of purifying selection, especially in humans and viral-sensing TLRs (Roach et al. 2005; Mukherjee et al. 2009; Barreiro et al. 2009), although evidence for balancing selection has also been demonstrated (Ferrer-Admetlla et al. 2008). Accordingly, in humans, TLR7 and TLR8 have been reported to be under strong purifying selection (e.g., Georgel et al. 2009; but see Areal et al. 2011). This is remarkable because both TLRs are responsible for the detection of ssRNA viruses and thus should be under positive selection as a result of an ongoing genetic arms race between host and pathogen and of coadaptation processes. Viral TLRs such as TLR7 might be constrained in accumulating structure-changing mutations that interfere with their recognition ability of viral peptides, because of their importance in virus recognition and autoimmunity. Non-viral TLRs, such as TLR4, can recognize a wider spectrum of pathogens and are therefore less restricted in accumulating mutations (Areal et al. 2011).

Initial studies in wildlife have revealed that levels of TLR diversity vary considerably not only among receptors but also among species (Fornůsková et al. 2013; Grueber et al. 2015). For an outbred species (in our study, at least in the continuous forest, landscape C) with such a large biogeographic range, the detected TLR genetic diversity is considered as being low. Cui et al. (2015a, b) have detected 12 TLR4 haplotypes and 4 TLR7 haplotypes in koalas. Grueber et al. (2015) have investigated several threatened bird species in New Zealand and reported 1–8 different TLR4 alleles and 1–2 TLR7 alleles per species. Two TLR4 haplotypes have been encountered in the water vole (*Arvicola amphibius*, Gavan et al. 2015). In species with a large biogeographic distribution, such as the European roe deer (*Capreolus capreolus*), 10 different TLR4 haplotypes were reported (Quéméré et al. 2015), and 59 TLR4 and 31 TLR7 alleles have so far been detected in humans (Barreiro et al. 2009). However, Whiteoak et al. (2018) discerned a low level of genetic diversity in the European badger (*Meles meles*) for TLR2 (three haplotypes) and TLR4 (one haplotype), together with a very low level of heterozygosity (<5%). Whether the low TLR diversity of *P. semispinosus* also holds true in other parts of its biogeographic range remains to be investigated, as does whether low levels of diversity are also present in other TLRs that have not been analyzed in our study but that might be of importance, such as TLR8, which is known to recognize ssRNA in humans (Heil et al. 2004). Moreover, studies using genome-wide SNPs will help to understand the population structure and demographics of the spiny rat populations and to place the observed low genetic diversity at TLRs in *P. semispinosus* into perspective.

### Association between TLR4 constitution, ecological & landscape factors, and nematode infection intensity

In *P. semispinosus*, we distinguished 14 different nematode morphotypes (0–6 morphotypes per individual). Up to seven different nematode morphotypes per species have been identified in a study on five small mammal species in the coastal rainforest Mata Atlântica of Brazil (Püttker et al. 2008). As in our study, the prevalence in rodents was very high (>94%), and individuals were infected by one or three different nematode morphotypes (Püttker et al. 2008). Froeschke et al. (2010) detected 15 helminth species (13 nematodes, 2 cestodes, 0–6 different species per individual) in a widely distributed rodent (striped mouse, *Rhabdomys pumilio*) sampled along a precipitation gradient from the Cape of South Africa to northern Namibia. In the long-tailed giant rat (*Leopoldamys sabanus*) in Borneo, 11 different helminth morphotypes have been identified (8 nematodes, 3 cestodes), with 0–4 morphotypes per individual (Lenz et al. 2009). In a rodent species (*Apodemus flavicollis*) living in temperate forests, eight different gastrointestinal nematode morphotypes, with 1–3 different nematode morphotypes per individual (Meyer-Lucht and Sommer 2009), were detected. Thus, some variation in the number of helminth species exists probably due to varying environmental conditions, and ecological and genetic differences of the host species. Given the importance of environmental factors in host–parasite interactions and parasite life history, it is anticipated that parasite loads will differ in landscapes affected by anthropogenic disturbance.

In our study, the nematode abundance differed among landscapes. Individuals from landscapes A and I showed the lowest burden of the most common nematode, whereas some of the other identified nematode morphotypes peaked in the human-modified study sites. Pathogen transmission and pressure in wildlife are contingent on multiple factors. In modified landscapes, pathogen spread can be influenced by the level of connectivity between habitat patches (Tanaka et al. 2014) and the mobility of the host species (Lechner et al. 2017). Pathogen transmission also depends on whether a directly transmitted pathogen or one that requires vectors is considered, and whether this is influenced by the existence of reservoir species other than the primary host (Ostfeld and Keesing 2012; Randolph and Dobson 2012). Pathogen prevalence is also likely to be affected by ecological variables that can be changed, among others, by anthropogenic disturbance. For instance, community traits such as species richness, assemblage pattern, and host population density are sensitive to habitat modifications and have been shown to be especially important in directly transmitted pathogens (Mills and Childs 1998; Woodroffe et al. 2012; Schmid et al. 2018). Accordingly, modified landscapes can harbor a higher or lower pathogen burden compared with the undisturbed habitat depending on the species and pathogens analyzed, thus emphasizing the complexity of disease dynamics (Becker and Zamudio 2011). The question is what drives the infection risk of wildlife, i.e., do ecological factors influenced by anthropogenic changes of the landscape, or immune genetic constitution (here at TLRs), or a combination explain better the infection probability of catching an infection?

In *P. semispinosus*, the best model explaining the variance in nematode loads included the haplotype TLR4_H1 and landscape as a surrogate for the most important influential ecological factors distinguishing the landscapes in our study, i.e. species diversity and host population density (see Schmid et al. 2018). Individuals carrying the TLR4_H1 were less intensely infected than those missing this haplotype. TLR4_Ht1 differed from the second common haplotype, TLR4_Ht2, by two SNPs, namely a synonymous SNP and a non-synonymous SNP, coding for alanine and valine, respectively, both of which are amino acids with hydrophobic side chains. The exchange of amino acids might interfere with binding probabilities with the pathogens, even though both fall within the same group of amino acids. The differences in the functional importance of TLR4 haplotypes might be attributable to the direct recognition of parasite-associated molecular patterns or might be an indirect association. TLRs can be activated and regulated by some helminthic species that are known to avoid recognition of the immune system through TLRs (Venugopal et al. 2008). Additionally, nematodes have been shown to harbor Gram-negative bacteria, such as *Wolbachia*, which could elicit a reaction of the innate immune system because of the interaction of the surface proteins from the bacteria with TLR4 (Brattig et al. 2004). Recognition of these particles by the TLRs could lead to inflammation in the host tissue, thus generating a more unsuitable environment for the parasites.

Comparable with our study, Lin et al. (2016) revealed contrasting effects of certain TLR4 haplotypes on nematode infection in sheep from New Zealand. Whereas certain haplotypes were associated with a lower nematode burden, others lead to a higher infection intensity. Gavan et al. (2015) linked various TLR4 genotypes to burdens of gamasid mites, fleas, and sheep tick larvae in a water vole (*A. amphibius*) population from northwest Scotland. The mechanism behind the difference in TLR4 distribution was attributed to parasite-mediated selection, a mechanism also possibly explaining the differences encountered in our study. Further studies have shown an association between TLR2 haplotypes and *Borrelia burgdorferi* in bank vole (*Myodes glareolus*) populations from Sweden (Tschirren et al. 2013). In contrast, different TLR11 and TLR12 haplotypes were not associated with *Toxoplasma gondii* infection in *Apodemus sylvaticus* (Morger et al. 2014).

### Association between TLR4 and TLR7 constitution, ecological & landscape factors, and *Hepacivirus* susceptibility

The *Hepaciviruses* encountered in the spiny rat belong to the genus *Hepacivirus* within the virus family Flaviviridae. The prototype virus is the HCV (species *Hepacivirus C*), which causes acute and chronic liver infections in humans (Simmonds et al. 2017). *Hepacivirus* are mainly transmitted by biting during aggressive behavior, which in turn is more pronounced in males than in females (Schmid et al. 2018). Both TLR4 and 7 have been shown to be associated with HCV infection in humans (Machida et al. 2006; Wang et al. 2011). An increase in TLR4 expression in HCV-infected humans has been attributed to the activation of the receptor through NS5A, a protein of the virus, and the presence of lipopolysaccharides, a compound of the cell wall of Gram-negative bacteria (Machida et al. 2006). Certain TLR7 variations occur with higher probability in individuals positive for HCV and additionally play a role in response to treatment with interferon-α. Moreover, sex-specific differences have been observed in humans and have been attributed to the location of the TLR7 gene on the X-chromosome (Schott et al. 2008). However, studies on the cause–response relationships between TLRs and viral pathogens in wildlife remain rare. Knafler et al. (2016) have shown a relationship between TLR3 and the beak and feather virus in red-crowned parakeets (*Cyanoramphus novaezelandiae*), but without further elaborating on the effect of specific haplotypes on infection status. Loots et al. (2018) reported a possible beneficial effect of a SNP in TLR2 on canine distemper virus susceptibility in South African lions, resulting in an amino acid exchange within the LRR.

Regarding TLR7, in spiny rats all genetic models had a lower AICc than the ecology & landscape or genetics & landscape model, and the delta AICc of the effects of the two identified haplotypes (TLR_Ht1, TLR_Ht2) and the heterozygous genotype was similar. The best model, however, did not assess a genetic variable as a significant contributor. Thus, contrary to our expectations, we detected no effect of the genetic constitution of TLR7 and the infection status with *Hepacivirus*. The high seroprevalence of the *Hepacivirus* within our study population might be an indicator for reduced clinical manifestation and the (currently) less severe impact on the health of the animals and, thus, selection pressure on this TLR. We have also observed no sex-specific differences, even though TLR7 is located on the X-chromosome in mice and, therefore, probably also in the spiny rat.

On the contray, an individual’s TLR4 constitution had a strong effect on *Hepacivirus* prevalence. All individuals carrying TLR4_Ht3 were detected as *Hepacivirus-*positive. Yet, the AICc of the model including TLR4_Ht3 was only slightly lower than the model including the other rare haplotype TLR4_Ht4 (delta AICc < 0.1). Individuals carrying TLR4_Ht4 were less infected in landscapes C and A than individuals carrying the haplotypes TLR4_Ht1, Ht2, or Ht3. Whereas the models including these specific TLR haplotypes explained differences in the prevalence of *Hepacivirus* infections, the ecology & landscape model resulted in a much lower AICc that was only topped by a combination of ecological and genetic factors at a landscape level (genetics & landscape model). TLR4 is known to detect a wide range of PAMPs, with the envelope proteins of human endogenous retroviruses activating the innate immune system through CD14/TLR4 (Rolland et al. 2006). TLR4_Ht1 and TLR4_Ht2 differ by one amino acid (alanine/valine), both of which belong to the amino acids with hydrophobic side chains. TLR4_Ht3 differed from the other three haplotypes by an acidic polar amino acid at position 234, and TLR4_Ht4 differed by a tryptophan instead of an arginine at position 185. This suggests that TLR4 haplotypes harbor different functional attributes in their ligand-binding capacities and, thus, in their response to selection pressures induced by, among others, nematode and *Hepacivirus* infections.

*Hepacivirus* prevalence was lowest in landscape A, probably because of shifts in species assemblages affecting population density and sex ratio (so-called “dilution effect”, Schmid et al. 2018). In landscape A, TLR4_Ht2 was more frequently encountered than in the landscapes C (continuous forest) and I (forested islands). Even though we were unable to detect any association of TLR4_Ht2 regarding the abundance of the gastrointestinal nematode and *Hepacivirus* infections, beneficial effects of this receptor with respect to other pathogens not investigated in the present study might explain this discrepancy. Possibly, the differences in the haplotype frequencies could be attributable to pathogen-driven selection promoted by pathogens that were only present in landscape A (vicinity to humans, domestic animals, and possible non-native species, e.g., *Rattus norvegicus*, Flacke et al. 2013) but not in landscapes C and I (both are protected, and no contact with humans, domestic animals, and possible non-native species exists). Due to statistical constraints, we were not able to investigate whether frequencies between landscapes also differ on the genotype level because some genotypes occurred at extremely low frequencies (0.63–4.43%). This particularly applies to combinations including the rare haplotypes (TLR4_Ht3 and TLR4_Ht4). Moreover, since the present study did not include neutral markers, we could not judge whether the differences in TLR haplotype frequencies across landscapes were due to chance or differences in selection acting in different landscapes.

In conclusion, we detected evidence for the advantage/disadvantage of carrying specific haplotypes in pathogen resistance, which is in agreement with the assumptions of a rare allele advantage/frequency-dependent selection. Individuals carrying the common TLR4_Ht1 haplotype were less intensely infected by the most abundant strongyle nematode. Individuals carrying the rare TLR4_Ht3 haplotype were all *Hepacivirus*-positive, where those carrying the rare haplotype TLR4_Ht4 were less often infected with *Hepacivirus* than individuals with other haplotypes. We further detected strong differences in the frequency distribution of haplotypes across human-modified landscapes together with differing pathogen pressures. Further studies using genome-wide SNP markers will help to judge whether differences in TLR haplotype frequencies across landscapes are due to genetic drift or differences in selection acting in different landscapes. Our study highlights the importance of considering immune genetic as well as ecological factors in order to understand the effects of anthropogenic changes on wildlife health. Since changes in pathogen pressures attributable to anthropogenic habitat modifications and a higher contact probability of wildlife, domestic animals, and humans are increasingly being reported, disentanglement of the mechanisms influencing susceptibility/resistance to infections is crucial for wildlife health and the prevention of potential spillovers from pathogen reservoirs causing zoonotic diseases.

## Data archiving

TLR sequences are archived with GenBank under accession numbers MT136926–MT136931.

## Supplementary information

Supplementary Tables 1_11

Supplementary Files 1_3

Supplementary Figures 1_3
