# Supplementary material for: Pathogen-associated selection on innate immunity genes (TLR4, TLR7) in a neotropical rodent in landscapes differing in anthropogenic disturbance
Source: Heredity (Edinb). 2020 Jul 2;125(4):184–99. doi: 10.1038/s41437-020-0331-y (PMC7490709; doi:10.1038/s41437-020-0331-y)
Supplement: Supplementary file 1 — Supplementary Tables 1_11 [file 41437_2020_331_MOESM1_ESM.pdf]

Table S1: GPS coordinates and number of individuals that were investigated at TLR4 and TLR7, in each study site of landscapes C, A, and I.

Landscape	GPS coordinates	N_{TLR4}	N_{TLR7}	$N_{Nematodes}$	$N_{TLR4\ \&\ Hepacivirus}$	$N_{TLR4\ \&\ Nematodes}$	$N_{TLR7\ \&\ Hepacivirus}$
**Landscape C**							
Study site 1	9°12'16.213"N 79°49'47.916"W	12	12	20	11	8	11
Study site 2	9°9'47.021"N 79°48'1.615"W	11	9	31	7	8	6
Study site 3	9°6'49.921"N 79°51'34.373"W	10	10	17	9	8	9
Study site 4	9°7'3.448"N 79°50'13.9"W	10	5	15	10	9	5
Study site 5	9°9'10.794"N 79°53'9.62"W	10	10	18	10	6	10
**Landscape A**							
Study site 1	9°15'53.156"N 79°39'24.327"W	10	0	22	9	4	0
Study site 2	9°15'26.963"N 79°39'21.652"W	10	0	11	10	7	0
Study site 3	9°16'42.757"N 79°39'21.364"W	20	3	22	20	11	3
Study site 4	9°14'54.512"N 79°42'11.008"W	3	1	4	3	3	1
Study site 5	9°15'30.407"N 79°42'28.91"W	9	5	11	9	8	5
**Landscape I**							
Study site 1	9°12'41.726"N 79°53'31.155"W	10	8	44	10	10	8
Study site 2	9°8'16.465"N 79°50'5.343"W	10	5	18	9	9	4

Study site 3	9°10'44.09"N 79°50'54.793"W	10	4	12	10	5	4
Study site 4	9°12'29.492"N 79°54'26.677"W	12	12	25	11	5	11
Study site 5	9°12'19.18"N 79°50'53.098"W	11	11	23	11	4	11

Table S2: Accession numbers of TLR4 and TLR7 sequences of the various mammals and birds used for primer design (marked with *) and to infer their phylogenetic TLR relationships (Supplementary Figure 1, Supplementary Figure 2).

Species	Common name	TLR4	TLR7
Ailuropoda melanoleuca	Giant panda	XM_002929889.2	XM_002915062.2
Apteryx australis mantelli	North Island brown kiwi	XM_013961606.1	XM_013950523.1
Bos Taurus	Cattle	NM_174198.6	NM_001033761.1
Bubalus bubalis	Water buffalo	NM_001290903.1	NM_001290938.1
Camelus dromedarius	Dromedary	XM_010998333.1	XM_010993639.1
Canis lupus	Gray wolf	NM_001002950.2	NM_001048124.2
Cavia porcellus	Guinea pig	*XM_003478841	*XM_003462893
Cercocebus atys	Sooty mangabey	NM_001305968.1	NM_001305955.1
Chinchilla lanigera	Long-tailed chinchilla	*XM_005382606	*XM_005411374
Condylura cristata	Star-nosed mole	XM_004677395.2	XM_004689851.2
Cyanoramphus novaezelandiae	Red-crowned parakeet	KP250074.1	KP250139.1
Dasypus novemcinctus	Nine-banded armadillo	XM_004463152.2	XM_012525391.1
Eptesicus fuscus	Big brown bat	XM_008152116.1	XM_008156577.1
Equus caballus	Horse	NM_001099769.2	NM_001081771.2
Equus zebra hartmannae	Hartmann's mountain zebra	JN797515.1	KC510088.1
Erinaceus europaeus	European hedgehog	XM_007521939.1	XM_007517472.1
Felis catus	Cat	NM_001009223.1	NM_001080133.1
Fukomys damarensis	Damaraland mole-rat	*XM_010639454	*XM_010613972
Galeopterus variegatus	Sunda flying lemur	XM_008587300.1	XM_008571332.1
Gallus gallus	Red junglefowl	FJ915520.1	FJ915592.1
Heterocephalus glaber	Naked mole-rat	*XM_004849478<br>XM_004849478.2	*XM_004871035<br>XM_004871035.1
Homo sapiens	Human	NM_138557.2	DQ022180.1
Ictidomys tridecemlineatus	Thirteen-lined ground squirrel	XM_013356765.1	XM_013356282.1
Lipotes vexillifer	Baiji	XM_007449712.1	XM_007455055.1
Loxodonta africana	African bush elephant	XM_003407779.2	XM_010595793.1

Macaca mulatta	Rhesus macaque	NM_001037092.1	NM_001130426.1
Microcebus murinus	Gray mouse lemur	XM_012768779.1	XM_012784818.1
Mus musculus	House mouse	NM_021297.3	NM_001290758.1
Myotis davidii	David's myotis	XM_006772885.1	XM_006763796.1
Octodon degus	Common degu	*XM_004641503 XM_004641503.2	*XM_004635044 XM_004635044.1
Odobenus rosmarus divergens	Walrus	XM_004416046.1	XM_012565512.1
Ornithorhynchus anatinus	Platypus	XM_007661855.1	XM_007670389.1
Otolemur garnettii	Northern greater galago	XM_012802373.1	XM_003793056.1
Ovis aries	Sheep	NM_001135930.1	NM_001135059.1
Pan troglodytes	Common chimpanzee	NM_001144863.1	NM_001130133.1
Panthera tigris altaica	Siberian tiger	XM_007094338.1	XM_007090078.1
Petroica australis australis	South Island robin	KP250089.1	JX502665.1
Phascolarctos cinereus	Koala	KP792551.1	KM816885.1
Physeter catodon	Sperm whale	AB500181.1	XM_007121207.1
Pongo abelii	Sumatran orangutan	XM_003777437.2	XM_003780383.2
Pteropus vampyrus	Large flying fox	XM_011360965.1	XM_011362910.1
Rattus nitidus	Himalayan field rat	KC811684.1	KC811782.1
Rattus norvegicus	Brown rat	NM_019178.1	NM_001097582.1
Rattus tanezumi	Tanezumi rat	KC811700.1	KC811799.1
Sarcophilus harrisii	Tasmanian devil	XM_003757076.2	XM_003765580.1
Sorex araneus	Common shrew	XM_004615682.1	XM_004612185.1
Strigops habroptilus	Kakapo	KF265300.1	KF265324.1
Sus scrofa	Wild boar	NM_001293316.1	NM_001097434.1
Tarsius syrichta	Philippine tarsier	XM_008053545.1	XM_008072445.1
Tupaia belangeri chinensis	Northern treeshrew	XM_006142796.2	XM_006172018.1
Ursus maritimus	Polar bear	XM_008698840.1	XM_008696372.1

Table S3: Relative abundance of haplotypes and genotypes and the location of variable positions in the overall data set of spiny rats (*P. semispinosus*). **A)** TLR4 **B)** TLR7. The variable TLR4 nucleotide positions 552, 553, 704, and 993 are numbered according to the TLR4 sequence of *Octodon degus* (XM_004641503.2). The number of the variable TLR7 nucleotide position is according to the TLR7 sequence of *Octodon degus* (XM_004635044.1). syn synonymous substitution. Sample sizes are provided in Table S1.

A

Haplotype/genotype	relAbund	552	553	704	993
TLR4_Ht1	0.72	T	C	T	A
TLR4_Ht2	0.76	G	C	C	A
TLR4_Ht3	0.05	G	C	A	G
TLR4_Ht4	0.10	T	T	T	A
Amino acid change		syn (Leu)	Arg/Trp	Ala/Val/Glu	Ile/Met
Ht1Ht1	0.15				
Ht1Ht2	0.49				
Ht1Ht3	0.02				
Ht1Ht4	0.04				
Ht2Ht2	0.22				
Ht2Ht3	0.01				
Ht2Ht4	0.04				
Ht3Ht3	0.01				
Ht3Ht4	0.01				
Ht4Ht4	0.01				

B

Haplotype/genotype	relAbund	1148
TLR7_Ht1	0.62	C
TLR7_Ht2	0.70	T
Amino acid change		syn (Asn)
Ht1	0.21	
Ht1Ht1	0.09	
Ht1Ht2	0.32	
Ht2	0.16	
Ht2Ht2	0.22	

Table S4: Best model (*Genetics & landscape model*) explaining the variance in infection intensity with the most common nematode (N= 105).

Variable	Estimate	Std. Error	z value	P	
(Intercept)	8.128	0.264	30.830	< 2e-16	***
TLR4_Ht1 (present)	-0.501	0.253	-1.978	0.048	*
Landscape (A)	-0.938	0.267	-3.513	<0.001	***
Landscape (I)	-0.557	0.263	-2.115	0.034	*

Table 5a: Best model (TLR4_Ht3 and landscape from the *Genetics & landscape model*) explaining the variance in *Hepacivirus* prevalence (n= 149).

	Estimate	Std. Error	z value	P	
(Intercept)	2.251	0.526	4.283	<0.001	***
Landscape (A)	-2.374	0.599	-3.966	<0.001	***
Landscape (I)	-0.054	0.706	-0.077	0.939	
TLR4_Ht3 (present)	16.314	1242.785	0.013	0.990	

Table 5b: Best model (TLR4_Ht4 and landscape from the *Genetics & landscape model*) explaining the variance in *Hepacivirus* prevalence (n= 149).

	Estimate	Std. Error	z value	P	
(Intercept)	2.571	0.549	4.682	<0.001	***
Landscape (A)	-2.458	0.606	-4.058	<0.001	***
Landscape (I)	-0.243	0.713	-0.341	0.733	
TLR4_Ht4 (present)	-1.208	0.661	-1.827	0.068	.

Table S6: Comparison of models explaining the association between TLR7 constitution, ecological & landscape factors, and *Hepacivirus* infection status (n= 88). Model were split into three different categories: 1. *Genetic models* investigate the effects of different TLR haplotypes and heterozygosity, 2. *Ecological & landscape model* investigate landscape, sex, age and host population density in interaction with sex ratio as factors (Schmid *et al*., 2018), and 3. *Genetics & landscape models* combine genetic factors from the *genetic models* with landscape as a surrogate for ecological factors. Best model in bold.

Models	Df	AICc	delta
1. Genetic models			
Heterozygosity	2	73.995	0.088
**TLR7_Ht1**	**2**	**73.907**	**0.000**
TLR7_Ht2	2	74.229	0.322
2. Ecological & landscape model			
Landscape, sex, age, density:sex ratio	6	75.149	1.242
3. Genetics & landscape models			
Heterozygosity, landscape	4	75.492	1.585
TLR7_Ht1, landscape	4	75.214	1.308
TLR7_Ht2, landscape	4	75.675	1.769

Table S7: Best model (*Genetic model*) explaining the variance in *Hepacivirus* prevalence in interaction with TLR7 (n=88).

	Estimate	Std. Error	z value	P	
(Intercept)	2.079	0.530	3.921	<0.001	***
TLR7_Ht1 (present)	-0.375	0.655	-0.572	0.567	

Table S8: Post-hoc test for factors of the best model (*Genetic model*) explaining the variance in *Hepacivirus* prevalence (N= 88).

Contrast	Ratio	SE	df	z ratio	P
TLR7_Ht1 (absent-present)	0.375	0.655	Inf	0.572	0.567

Table S9: Comparison of the *Ecological & landscape model* explaining nematode infection intensity (n= 105) without (Table 1) and with season as additional factor. Delta: AIC difference to the best model (Table 1).

Ecological & landscape model	df	AICc	delta
Landscape, sex, age, density:sex ratio	7	1756.384	7.511
Landscape, sex, age, density:sex ratio, season	8	1757.947	9.074

Table S10: Comparison of the *Ecological & landscape model* explaining *Hepacivirus* infection status (n= 149) without (Table 3) and with season as additional factor. Delta: AIC difference to the best model (Table 3).

Ecological & landscape model	df	AICc	Delta
Landscape, sex, age, density:sex ratio	7	136.798	1.850
Landscape, sex, age, density:sex ratio, season	8	136.750	1.802

Table S11: Comparison of the *Ecological & landscape model* explaining *Hepacivirus* infection status (n= 88) without (Table S6) and with season as additional factor. Delta: AIC difference to the best model (Table S6).

Ecological & landscape model	df	AICc	delta
Landscape, sex, age, density:sex ratio	7	75.149	1.242
Landscape, sex, age, density:sex ratio, season	8	74.615	0.708
