# Supplementary material for: Pathogen-associated selection on innate immunity genes (TLR4, TLR7) in a neotropical rodent in landscapes differing in anthropogenic disturbance
Source: Heredity (Edinb). 2020 Jul 2;125(4):184–99. doi: 10.1038/s41437-020-0331-y (PMC7490709; doi:10.1038/s41437-020-0331-y)

Pathogen-associated selection on innate immunity genes (TLR4, TLR7) in a neotropical rodent in landscapes differing in anthropogenic disturbance

Alexander Christoph Heni, Julian Schmid, Andrea Rasche, Victor Max Corman, Christian Drosten, Simone Sommer

Supplementary File 1: Details of the sampled individuals (i.e. sampling location, TLR haplotypes, infection status, sex, age).

Individual ID	Landscape	Study Site	Season	Sex	Age	FEC Nematode 3	Hepacivirus Prevalence	Density	Sex Ratio	TLR4 Genotype	TLR7 Genotype	TLR4_Ht1	TLR4_Ht2	TLR4_Ht3	TLR4_Ht4	TLR7_Ht1	TLR4_Ht2	TLR4 Heterozygosity	TLR7 Heterozygosity
1	C	1	1	M	A	3600	1	1.11	0.75	Prse_TLR4_Ht2.Prse_TLR4_Ht3	Prse_TLR7_Ht2	0	1	1	0	0	1	1	0
2	C	1	1	F	A	NA	1	1.11	0.75	Prse_TLR4_Ht1.Prse_TLR4_Ht2	Prse_TLR7_Ht2.Prse_TLR7_Ht2	1	1	0	0	0	1	1	0
3	C	1	1	M	A	4600	1	1.11	0.75	Prse_TLR4_Ht1.Prse_TLR4_Ht1	Prse_TLR7_Ht2	1	0	0	0	0	1	0	0
4	C	1	1	F	A	NA	1	1.11	0.75	Prse_TLR4_Ht3.Prse_TLR4_Ht3	Prse_TLR7_Ht1.Prse_TLR7_Ht2	0	0	1	0	1	1	0	1
5	C	1	1	F	A	800	1	1.11	0.75	Prse_TLR4_Ht2.Prse_TLR4_Ht2	Prse_TLR7_Ht2.Prse_TLR7_Ht2	0	1	0	0	0	1	0	0
6	C	1	1	M	A	500	NA	1.11	0.75	Prse_TLR4_Ht2.Prse_TLR4_Ht2	Prse_TLR7_Ht1	0	1	0	0	1	0	0	0
7	C	1	1	F	A	2000	1	1.11	0.75	Prse_TLR4_Ht1.Prse_TLR4_Ht2	Prse_TLR7_Ht1.Prse_TLR7_Ht1	1	1	0	0	1	0	1	0
8	C	1	2	F	A	6400	1	3.32	1.63	Prse_TLR4_Ht2.Prse_TLR4_Ht2	Prse_TLR7_Ht1.Prse_TLR7_Ht1	0	1	0	0	1	0	0	0
9	C	1	2	M	A	2100	1	3.32	1.63	Prse_TLR4_Ht1.Prse_TLR4_Ht2	Prse_TLR7_Ht1	1	1	0	0	1	0	1	0
10	C	1	2	F	A	NA	1	3.32	1.63	Prse_TLR4_Ht1.Prse_TLR4_Ht2	Prse_TLR7_Ht2.Prse_TLR7_Ht2	1	1	0	0	0	1	1	0
11	C	1	2	F	A	6500	1	3.32	1.63	Prse_TLR4_Ht2.Prse_TLR4_Ht2	Prse_TLR7_Ht2.Prse_TLR7_Ht2	0	1	0	0	0	1	0	0
12	C	1	2	M	J	NA	1	3.32	1.63	Prse_TLR4_Ht1.Prse_TLR4_Ht2	Prse_TLR7_Ht1	1	1	0	0	1	0	1	0
13	C	2	1	F	A	5450	NA	3.49	0.83	Prse_TLR4_Ht1.Prse_TLR4_Ht2	Prse_TLR7_Ht1.Prse_TLR7_Ht2	1	1	0	0	1	1	1	1
14	C	2	1	F	A	1750	1	3.49	0.83	Prse_TLR4_Ht2.Prse_TLR4_Ht4	Prse_TLR7_Ht2.Prse_TLR7_Ht2	0	1	0	1	0	1	1	0
15	C	2	1			NA	NA	3.49	0.83	Prse_TLR4_Ht1.Prse_TLR4_Ht2	Prse_TLR7_Ht1.Prse_TLR7_Ht2	1	1	0	0	1	1	1	1
16	C	2	1	M	A	NA	1	3.49	0.83	Prse_TLR4_Ht1.Prse_TLR4_Ht2	Prse_TLR7_Ht1	1	1	0	0	1	0	1	0
17	C	2	1	M	A	NA	1	3.49	0.83	Prse_TLR4_Ht1.Prse_TLR4_Ht4	Prse_TLR7_Ht1	1	0	0	1	1	0	1	0
18	C	2	1	M	A	5150	1	3.49	0.83	Prse_TLR4_Ht1.Prse_TLR4_Ht2	Prse_TLR7_Ht2	1	1	0	0	0	1	1	0

19	C	2	1	F	A	1050	1	3.49	0.83	Prse_TLR4_Ht1.Prse_TLR4_Ht2	Prse_TLR7_Ht1.Prse_TLR7_Ht2	1	1	0	0	1	1	1	1
20	C	2	1	F	A	2000	NA	3.49	0.83	Prse_TLR4_Ht1.Prse_TLR4_Ht2	Prse_TLR7_Ht1.Prse_TLR7_Ht2	1	1	0	0	1	1	1	1
21	C	2	1	F	A	1550	1	3.49	0.83	Prse_TLR4_Ht1.Prse_TLR4_Ht3	Prse_TLR7_Ht1.Prse_TLR7_Ht2	1	0	1	0	1	1	1	1
22	C	2	1	F	A	2750	NA	3.49	0.83	Prse_TLR4_Ht1.Prse_TLR4_Ht2	NA	1	1	0	0	NA	NA	1	NA
23	C	2	2	M	J	2550	1	11.48	1.33	Prse_TLR4_Ht1.Prse_TLR4_Ht1	NA	1	0	0	0	NA	NA	0	NA
24	C	3	1	F	A	3200	1	2.11	0.5	Prse_TLR4_Ht1.Prse_TLR4_Ht2	Prse_TLR7_Ht2.Prse_TLR7_Ht2	1	1	0	0	0	1	1	0
25	C	3	1	F	A	NA	1	2.11	0.5	Prse_TLR4_Ht1.Prse_TLR4_Ht2	Prse_TLR7_Ht1.Prse_TLR7_Ht2	1	1	0	0	1	1	1	1
26	C	3	1	F	A	1700	1	2.11	0.5	Prse_TLR4_Ht1.Prse_TLR4_Ht2	Prse_TLR7_Ht2.Prse_TLR7_Ht2	1	1	0	0	0	1	1	0
27	C	3	1	F	A	3700	1	2.11	0.5	Prse_TLR4_Ht1.Prse_TLR4_Ht2	Prse_TLR7_Ht2.Prse_TLR7_Ht2	1	1	0	0	0	1	1	0
28	C	3	1	F	A	300	1	2.11	0.5	Prse_TLR4_Ht1.Prse_TLR4_Ht1	Prse_TLR7_Ht1.Prse_TLR7_Ht1	1	0	0	0	1	0	0	0
29	C	3	1	F	J	NA	1	2.11	0.5	Prse_TLR4_Ht1.Prse_TLR4_Ht1	Prse_TLR7_Ht2.Prse_TLR7_Ht2	1	0	0	0	0	1	0	0
30	C	3	1	M	A	700	1	2.11	0.5	Prse_TLR4_Ht1.Prse_TLR4_Ht1	Prse_TLR7_Ht2	1	0	0	0	0	1	0	0
31	C	3	1	M	A	1150	1	2.11	0.5	Prse_TLR4_Ht1.Prse_TLR4_Ht1	Prse_TLR7_Ht1	1	0	0	0	1	0	0	0
32	C	3	1	F	A	600	NA	2.11	0.5	Prse_TLR4_Ht1.Prse_TLR4_Ht2	Prse_TLR7_Ht1.Prse_TLR7_Ht2	1	1	0	0	1	1	1	1
33	C	3	2	F	A	600	1	0.99	1.67	Prse_TLR4_Ht1.Prse_TLR4_Ht2	Prse_TLR7_Ht2.Prse_TLR7_Ht2	1	1	0	0	0	1	1	0
34	C	4	1	F	A	350	1	0.87	1	Prse_TLR4_Ht2.Prse_TLR4_Ht2	Prse_TLR7_Ht1.Prse_TLR7_Ht2	0	1	0	0	1	1	0	1
35	C	4	1	F	A	650	0	0.87	1	Prse_TLR4_Ht1.Prse_TLR4_Ht2	Prse_TLR7_Ht2.Prse_TLR7_Ht2	1	1	0	0	0	1	1	0
36	C	4	1	F	A	950	1	0.87	1	Prse_TLR4_Ht1.Prse_TLR4_Ht2	Prse_TLR7_Ht1.Prse_TLR7_Ht2	1	1	0	0	1	1	1	1
37	C	4	1	M	A	NA	1	0.87	1	Prse_TLR4_Ht1.Prse_TLR4_Ht1	Prse_TLR7_Ht2	1	0	0	0	0	1	0	0
38	C	4	1	M	A	1000	1	0.87	1	Prse_TLR4_Ht1.Prse_TLR4_Ht2	Prse_TLR7_Ht1	1	1	0	0	1	0	1	0
39	C	4	2	M	A	350	1	1.45	1	Prse_TLR4_Ht1.Prse_TLR4_Ht1	NA	1	0	0	0	NA	NA	0	NA
40	C	4	2	M	A	3625	1	1.45	1	Prse_TLR4_Ht1.Prse_TLR4_Ht2	NA	1	1	0	0	NA	NA	1	NA
41	C	4	2	F	A	2850	1	1.45	1	Prse_TLR4_Ht1.Prse_TLR4_Ht2	NA	1	1	0	0	NA	NA	1	NA
42	C	4	2	F	A	900	1	1.45	1	Prse_TLR4_Ht1.Prse_TLR4_Ht2	NA	1	1	0	0	NA	NA	1	NA
43	C	4	2	M	A	3425	1	1.45	1	Prse_TLR4_Ht1.Prse_TLR4_Ht2	NA	1	1	0	0	NA	NA	1	NA
44	C	5	1	F	A	200	1	2.04	0.75	Prse_TLR4_Ht2.Prse_TLR4_Ht2	Prse_TLR7_Ht1.Prse_TLR7_Ht1	0	1	0	0	1	0	0	0
45	C	5	1	F	A	NA	1	2.04	0.75	Prse_TLR4_Ht1.Prse_TLR4_Ht2	Prse_TLR7_Ht1.Prse_TLR7_Ht2	1	1	0	0	1	1	1	1
46	C	5	1	F	A	NA	0	2.04	0.75	Prse_TLR4_Ht1.Prse_TLR4_Ht4	Prse_TLR7_Ht2	1	0	0	1	0	1	1	0

47	C	5	1	M	A	4450	1	2.04	0.75	Prse_TLR4_Ht1.Prse_TLR4_Ht3	Prse_TLR7_Ht2	1	0	1	0	0	1	1	0
48	C	5	1	M	A	1850	1	2.04	0.75	Prse_TLR4_Ht4.Prse_TLR4_Ht4	Prse_TLR7_Ht1	0	0	0	1	1	0	0	0
49	C	5	1	M	A	NA	0	2.04	0.75	Prse_TLR4_Ht1.Prse_TLR4_Ht4	Prse_TLR7_Ht1	1	0	0	1	1	0	1	0
50	C	5	1	F	A	4200	0	2.04	0.75	Prse_TLR4_Ht1.Prse_TLR4_Ht2	Prse_TLR7_Ht1.Prse_TLR7_Ht2	1	1	0	0	1	1	1	1
51	C	5	1	M	A	NA	1	2.04	0.75	Prse_TLR4_Ht3.Prse_TLR4_Ht3	Prse_TLR7_Ht1	0	0	1	0	1	0	0	0
52	C	5	1	F	A	900	1	2.04	0.75	Prse_TLR4_Ht1.Prse_TLR4_Ht1	Prse_TLR7_Ht2.Prse_TLR7_Ht2	1	0	0	0	0	1	0	0
53	C	5	2	F	A	2350	1	2.34	0.78	Prse_TLR4_Ht1.Prse_TLR4_Ht1	Prse_TLR7_Ht1.Prse_TLR7_Ht1	1	0	0	0	1	0	0	0
54	A	1	2	F	J	2500	1	6.75	0.62	Prse_TLR4_Ht2.Prse_TLR4_Ht2	NA	0	1	0	0	NA	NA	0	NA
55	A	1	2	M	A	1700	1	6.75	0.62	Prse_TLR4_Ht1.Prse_TLR4_Ht2	NA	1	1	0	0	NA	NA	1	NA
56	A	1	2	F	A	4450	1	6.75	0.62	Prse_TLR4_Ht3.Prse_TLR4_Ht4	NA	0	0	1	1	NA	NA	1	NA
57	A	1	2	M	J	3350	0	6.75	0.62	Prse_TLR4_Ht1.Prse_TLR4_Ht2	NA	1	1	0	0	NA	NA	1	NA
58	A	1	2	F	J	NA	1	6.75	0.62	Prse_TLR4_Ht1.Prse_TLR4_Ht2	NA	1	1	0	0	NA	NA	1	NA
59	A	1	2	M	J	NA	0	6.75	0.62	Prse_TLR4_Ht2.Prse_TLR4_Ht2	NA	0	1	0	0	NA	NA	0	NA
60	A	1	2	F	J	NA	0	6.75	0.62	Prse_TLR4_Ht1.Prse_TLR4_Ht2	NA	1	1	0	0	NA	NA	1	NA
61	A	1	2	M	J	NA	1	6.75	0.62	Prse_TLR4_Ht2.Prse_TLR4_Ht2	NA	0	1	0	0	NA	NA	0	NA
62	A	1	2	F	J	NA	1	6.75	0.62	Prse_TLR4_Ht2.Prse_TLR4_Ht4	NA	0	1	0	1	NA	NA	1	NA
63	A	1	2	M	J	NA	NA	6.75	0.62	Prse_TLR4_Ht1.Prse_TLR4_Ht2	NA	1	1	0	0	NA	NA	1	NA
64	A	2	2	M	A	NA	1	2.53	1.29	Prse_TLR4_Ht2.Prse_TLR4_Ht2	NA	0	1	0	0	NA	NA	0	NA
65	A	2	2	M	A	1600	1	2.53	1.29	Prse_TLR4_Ht2.Prse_TLR4_Ht2	NA	0	1	0	0	NA	NA	0	NA
66	A	2	2	F	A	800	0	2.53	1.29	Prse_TLR4_Ht1.Prse_TLR4_Ht4	NA	1	0	0	1	NA	NA	1	NA
67	A	2	2	M	A	600	0	2.53	1.29	Prse_TLR4_Ht2.Prse_TLR4_Ht4	NA	0	1	0	1	NA	NA	1	NA
68	A	2	2	M	A	1100	0	2.53	1.29	Prse_TLR4_Ht1.Prse_TLR4_Ht2	NA	1	1	0	0	NA	NA	1	NA
69	A	2	2	M	A	500	1	2.53	1.29	Prse_TLR4_Ht2.Prse_TLR4_Ht3	NA	0	1	1	0	NA	NA	1	NA
70	A	2	2	F	A	NA	0	2.53	1.29	Prse_TLR4_Ht1.Prse_TLR4_Ht2	NA	1	1	0	0	NA	NA	1	NA
71	A	2	2	F	J	NA	0	2.53	1.29	Prse_TLR4_Ht1.Prse_TLR4_Ht1	NA	1	0	0	0	NA	NA	0	NA
72	A	2	2	F	J	400	0	2.53	1.29	Prse_TLR4_Ht1.Prse_TLR4_Ht1	NA	1	0	0	0	NA	NA	0	NA
73	A	2	2	F	A	750	1	2.53	1.29	Prse_TLR4_Ht1.Prse_TLR4_Ht2	NA	1	1	0	0	NA	NA	1	NA
74	A	3	2	M	A	1200	1	4.64	0.72	Prse_TLR4_Ht1.Prse_TLR4_Ht2	NA	1	1	0	0	NA	NA	1	NA

75	A	3	2	F	J	1800	0	4.64	0.72	Prse_TLR4_Ht1.Prse_TLR4_Ht2	NA	1	1	0	0	NA	NA	1	NA
76	A	3	2	M	A	600	1	4.64	0.72	Prse_TLR4_Ht1.Prse_TLR4_Ht1	NA	1	0	0	0	NA	NA	0	NA
77	A	3	2	F	A	800	0	4.64	0.72	Prse_TLR4_Ht1.Prse_TLR4_Ht2	NA	1	1	0	0	NA	NA	1	NA
78	A	3	2	M	A	950	0	4.64	0.72	Prse_TLR4_Ht2.Prse_TLR4_Ht2	NA	0	1	0	0	NA	NA	0	NA
79	A	3	2	F	A	NA	1	4.64	0.72	Prse_TLR4_Ht2.Prse_TLR4_Ht2	NA	0	1	0	0	NA	NA	0	NA
80	A	3	2	F	A	600	0	4.64	0.72	Prse_TLR4_Ht2.Prse_TLR4_Ht4	NA	0	1	0	1	NA	NA	1	NA
81	A	3	2	M	A	350	0	4.64	0.72	Prse_TLR4_Ht1.Prse_TLR4_Ht2	NA	1	1	0	0	NA	NA	1	NA
82	A	3	2	F	A	250	0	4.64	0.72	Prse_TLR4_Ht1.Prse_TLR4_Ht4	NA	1	0	0	1	NA	NA	1	NA
83	A	3	2	F	A	900	1	4.64	0.72	Prse_TLR4_Ht2.Prse_TLR4_Ht2	NA	0	1	0	0	NA	NA	0	NA
84	A	3	2	F	J	NA	0	4.64	0.72	Prse_TLR4_Ht2.Prse_TLR4_Ht2	NA	0	1	0	0	NA	NA	0	NA
85	A	3	2	F	A	NA	1	4.64	0.72	Prse_TLR4_Ht2.Prse_TLR4_Ht2	Prse_TLR7_Ht2.Prse_TLR7_Ht2	0	1	0	0	0	1	0	0
86	A	3	2	F	J	NA	0	4.64	0.72	Prse_TLR4_Ht2.Prse_TLR4_Ht2	NA	0	1	0	0	NA	NA	0	NA
87	A	3	2	F	A	NA	0	4.64	0.72	Prse_TLR4_Ht2.Prse_TLR4_Ht4	NA	0	1	0	1	NA	NA	1	NA
88	A	3	2	F	J	NA	0	4.64	0.72	Prse_TLR4_Ht1.Prse_TLR4_Ht2	NA	1	1	0	0	NA	NA	1	NA
89	A	3	2	M	A	150	1	4.64	0.72	Prse_TLR4_Ht2.Prse_TLR4_Ht2	Prse_TLR7_Ht2	0	1	0	0	0	1	0	0
90	A	3	2	M	J	NA	0	4.64	0.72	Prse_TLR4_Ht2.Prse_TLR4_Ht2	NA	0	1	0	0	NA	NA	0	NA
91	A	3	2	M	J	NA	0	4.64	0.72	Prse_TLR4_Ht2.Prse_TLR4_Ht2	NA	0	1	0	0	NA	NA	0	NA
92	A	3	2	M	J	NA	0	4.64	0.72	Prse_TLR4_Ht1.Prse_TLR4_Ht2	NA	1	1	0	0	NA	NA	1	NA
93	A	3	2	M	A	350	1	4.64	0.72	Prse_TLR4_Ht1.Prse_TLR4_Ht1	Prse_TLR7_Ht1	1	0	0	0	1	0	0	0
94	A	4	1	F	A	0	1	0.75	1	Prse_TLR4_Ht2.Prse_TLR4_Ht2	NA	0	1	0	0	NA	NA	0	NA
95	A	4	1	M	A	400	1	0.75	1	Prse_TLR4_Ht1.Prse_TLR4_Ht2	Prse_TLR7_Ht2	1	1	0	0	0	1	1	0
96	A	4	2	M	A	100	0	0.75	1	Prse_TLR4_Ht1.Prse_TLR4_Ht1	NA	1	0	0	0	NA	NA	0	NA
97	A	5	1	F	A	150	1	0.87	0.5	Prse_TLR4_Ht1.Prse_TLR4_Ht2	Prse_TLR7_Ht1.Prse_TLR7_Ht2	1	1	0	0	1	1	1	1
98	A	5	1	F	A	2050	0	0.87	0.5	Prse_TLR4_Ht2.Prse_TLR4_Ht2	Prse_TLR7_Ht1.Prse_TLR7_Ht2	0	1	0	0	1	1	0	1
99	A	5	1	F	A	4200	0	0.87	0.5	Prse_TLR4_Ht2.Prse_TLR4_Ht2	Prse_TLR7_Ht1.Prse_TLR7_Ht1	0	1	0	0	1	0	0	0
100	A	5	1	F	A	0	0	0.87	0.5	Prse_TLR4_Ht1.Prse_TLR4_Ht2	Prse_TLR7_Ht2.Prse_TLR7_Ht2	1	1	0	0	0	1	1	0
101	A	5	1	M	A	NA	1	0.87	0.5	Prse_TLR4_Ht1.Prse_TLR4_Ht2	Prse_TLR7_Ht2	1	1	0	0	0	1	1	0
102	A	5	2	F	A	100	1	1.02	0.75	Prse_TLR4_Ht2.Prse_TLR4_Ht2	NA	0	1	0	0	NA	NA	0	NA

103	A	5	2	F	A	1150	1	1.02	0.75	Prse_TLR4_Ht2.Prse_TLR4_Ht2	NA	0	1	0	0	NA	NA	0	NA
104	A	5	2	M	A	350	1	1.02	0.75	Prse_TLR4_Ht1.Prse_TLR4_Ht2	NA	1	1	0	0	NA	NA	1	NA
105	A	5	2	M	A	100	1	1.02	0.75	Prse_TLR4_Ht1.Prse_TLR4_Ht1	NA	1	0	0	0	NA	NA	0	NA
106	I	1	1	F	A	2250	1	11.54	0.45	Prse_TLR4_Ht1.Prse_TLR4_Ht2	Prse_TLR7_Ht2.Prse_TLR7_Ht2	1	1	0	0	0	1	1	0
107	I	1	1	F	A	650	1	11.54	0.45	Prse_TLR4_Ht1.Prse_TLR4_Ht2	Prse_TLR7_Ht1.Prse_TLR7_Ht2	1	1	0	0	1	1	1	1
108	I	1	1	F	A	300	1	11.54	0.45	Prse_TLR4_Ht1.Prse_TLR4_Ht2	Prse_TLR7_Ht1.Prse_TLR7_Ht2	1	1	0	0	1	1	1	1
109	I	1	1	F	A	0	1	11.54	0.45	Prse_TLR4_Ht1.Prse_TLR4_Ht2	Prse_TLR7_Ht2.Prse_TLR7_Ht2	1	1	0	0	0	1	1	0
110	I	1	1	F	A	0	0	11.54	0.45	Prse_TLR4_Ht1.Prse_TLR4_Ht1	Prse_TLR7_Ht2.Prse_TLR7_Ht2	1	0	0	0	0	1	0	0
111	I	1	1	M	J	350	1	11.54	0.45	Prse_TLR4_Ht1.Prse_TLR4_Ht2	Prse_TLR7_Ht2	1	1	0	0	0	1	1	0
112	I	1	1	F	A	50	1	11.54	0.45	Prse_TLR4_Ht1.Prse_TLR4_Ht2	Prse_TLR7_Ht2.Prse_TLR7_Ht2	1	1	0	0	0	1	1	0
113	I	1	1	F	A	100	1	11.54	0.45	Prse_TLR4_Ht1.Prse_TLR4_Ht1	Prse_TLR7_Ht2.Prse_TLR7_Ht2	1	0	0	0	0	1	0	0
114	I	1	2	M	A	1250	1	22.25	0.58	Prse_TLR4_Ht1.Prse_TLR4_Ht1	NA	1	0	0	0	NA	NA	0	NA
115	I	1	2	M	A	150	1	22.25	0.58	Prse_TLR4_Ht1.Prse_TLR4_Ht2	NA	1	1	0	0	NA	NA	1	NA
116	I	2	1	F	A	1700	NA	6.34	0.9	Prse_TLR4_Ht1.Prse_TLR4_Ht1	Prse_TLR7_Ht1.Prse_TLR7_Ht2	1	0	0	0	1	1	0	1
117	I	2	1	F	A	50	0	6.34	0.9	Prse_TLR4_Ht1.Prse_TLR4_Ht2	Prse_TLR7_Ht1.Prse_TLR7_Ht2	1	1	0	0	1	1	1	1
118	I	2	1	M	A	600	1	6.34	0.9	Prse_TLR4_Ht1.Prse_TLR4_Ht1	Prse_TLR7_Ht2	1	0	0	0	0	1	0	0
119	I	2	1	F	J	350	0	6.34	0.9	Prse_TLR4_Ht1.Prse_TLR4_Ht2	Prse_TLR7_Ht1.Prse_TLR7_Ht2	1	1	0	0	1	1	1	1
120	I	2	1	F	J	50	1	6.34	0.9	Prse_TLR4_Ht1.Prse_TLR4_Ht2	Prse_TLR7_Ht1.Prse_TLR7_Ht2	1	1	0	0	1	1	1	1
121	I	2	2	F	A	3425	1	2.02	0.17	Prse_TLR4_Ht2.Prse_TLR4_Ht2	NA	0	1	0	0	NA	NA	0	NA
122	I	2	2	F	A	2575	1	2.02	0.17	Prse_TLR4_Ht1.Prse_TLR4_Ht2	NA	1	1	0	0	NA	NA	1	NA
123	I	2	2	F	A	1709	1	2.02	0.17	Prse_TLR4_Ht1.Prse_TLR4_Ht2	NA	1	1	0	0	NA	NA	1	NA
124	I	2	2	M	A	NA	1	2.02	0.17	Prse_TLR4_Ht2.Prse_TLR4_Ht2	NA	0	1	0	0	NA	NA	0	NA
125	I	2	2	F	A	400	1	2.02	0.17	Prse_TLR4_Ht1.Prse_TLR4_Ht2	NA	1	1	0	0	NA	NA	1	NA
126	I	3	1	M	A	400	1	0.7	1.5	Prse_TLR4_Ht1.Prse_TLR4_Ht2	Prse_TLR7_Ht1	1	1	0	0	1	0	1	0
127	I	3	1	M	A	1500	1	0.7	1.5	Prse_TLR4_Ht1.Prse_TLR4_Ht2	Prse_TLR7_Ht1	1	1	0	0	1	0	1	0
128	I	3	1	M	A	1250	1	0.7	1.5	Prse_TLR4_Ht1.Prse_TLR4_Ht2	Prse_TLR7_Ht1	1	1	0	0	1	0	1	0
129	I	3	1	F	A	NA	1	0.7	1.5	Prse_TLR4_Ht2.Prse_TLR4_Ht2	Prse_TLR7_Ht1.Prse_TLR7_Ht1	0	1	0	0	1	0	0	0
130	I	3	2	M	A	4100	1	2.25	0.78	Prse_TLR4_Ht1.Prse_TLR4_Ht2	NA	1	1	0	0	NA	NA	1	NA

131	I	3	2	F	J	NA	1	2.25	0.78	Prse_TLR4_Ht1.Prse_TLR4_Ht2	NA	1	1	0	0	NA	NA	1	NA
132	I	3	2	M	A	NA	1	2.25	0.78	Prse_TLR4_Ht1.Prse_TLR4_Ht2	NA	1	1	0	0	NA	NA	1	NA
133	I	3	2	F	J	NA	1	2.25	0.78	Prse_TLR4_Ht1.Prse_TLR4_Ht2	NA	1	1	0	0	NA	NA	1	NA
134	I	3	2	F	A	700	1	2.25	0.78	Prse_TLR4_Ht1.Prse_TLR4_Ht2	NA	1	1	0	0	NA	NA	1	NA
135	I	3	2	F	A	NA	1	2.25	0.78	Prse_TLR4_Ht1.Prse_TLR4_Ht2	NA	1	1	0	0	NA	NA	1	NA
136	I	4	1	F	A	450	1	7.4	0.63	Prse_TLR4_Ht1.Prse_TLR4_Ht2	Prse_TLR7_Ht1.Prse_TLR7_Ht2	1	1	0	0	1	1	1	1
137	I	4	1	F	A	NA	0	7.4	0.63	Prse_TLR4_Ht1.Prse_TLR4_Ht2	Prse_TLR7_Ht1.Prse_TLR7_Ht1	1	1	0	0	1	0	1	0
138	I	4	1	F	A	NA	1	7.4	0.63	Prse_TLR4_Ht1.Prse_TLR4_Ht2	Prse_TLR7_Ht1.Prse_TLR7_Ht2	1	1	0	0	1	1	1	1
139	I	4	1	F	A	850	1	7.4	0.63	Prse_TLR4_Ht2.Prse_TLR4_Ht2	Prse_TLR7_Ht2.Prse_TLR7_Ht2	0	1	0	0	0	1	0	0
140	I	4	1	F	A	1150	1	7.4	0.63	Prse_TLR4_Ht1.Prse_TLR4_Ht2	Prse_TLR7_Ht1.Prse_TLR7_Ht2	1	1	0	0	1	1	1	1
141	I	4	1	F	A	1800	1	7.4	0.63	Prse_TLR4_Ht1.Prse_TLR4_Ht4	Prse_TLR7_Ht1.Prse_TLR7_Ht2	1	0	0	1	1	1	1	1
142	I	4	1	M	A	NA	0	7.4	0.63	Prse_TLR4_Ht2.Prse_TLR4_Ht2	Prse_TLR7_Ht1	0	1	0	0	1	0	0	0
143	I	4	1	F	A	750	1	7.4	0.63	Prse_TLR4_Ht2.Prse_TLR4_Ht2	Prse_TLR7_Ht2.Prse_TLR7_Ht2	0	1	0	0	0	1	0	0
144	I	4	1	F	A	NA	NA	7.4	0.63	Prse_TLR4_Ht1.Prse_TLR4_Ht2	Prse_TLR7_Ht1.Prse_TLR7_Ht2	1	1	0	0	1	1	1	1
145	I	4	1	M	A	NA	1	7.4	0.63	Prse_TLR4_Ht1.Prse_TLR4_Ht1	Prse_TLR7_Ht2	1	0	0	0	0	1	0	0
146	I	4	1	M	A	NA	1	7.4	0.63	Prse_TLR4_Ht2.Prse_TLR4_Ht4	Prse_TLR7_Ht1	0	1	0	1	1	0	1	0
147	I	4	1	F	A	NA	1	7.4	0.63	Prse_TLR4_Ht1.Prse_TLR4_Ht4	Prse_TLR7_Ht1.Prse_TLR7_Ht2	1	0	0	1	1	1	1	1
148	I	5	1	F	A	3850	1	11.4	0.66	Prse_TLR4_Ht2.Prse_TLR4_Ht2	Prse_TLR7_Ht1.Prse_TLR7_Ht2	0	1	0	0	1	1	0	1
149	I	5	1	M	A	200	1	11.4	0.66	Prse_TLR4_Ht1.Prse_TLR4_Ht2	Prse_TLR7_Ht1	1	1	0	0	1	0	1	0
150	I	5	1	M	A	NA	1	11.4	0.66	Prse_TLR4_Ht1.Prse_TLR4_Ht2	Prse_TLR7_Ht2	1	1	0	0	0	1	1	0
151	I	5	1	F	A	NA	1	11.4	0.66	Prse_TLR4_Ht1.Prse_TLR4_Ht1	Prse_TLR7_Ht1.Prse_TLR7_Ht2	1	0	0	0	1	1	0	1
152	I	5	1	F	A	NA	1	11.4	0.66	Prse_TLR4_Ht1.Prse_TLR4_Ht3	Prse_TLR7_Ht1.Prse_TLR7_Ht2	1	0	1	0	1	1	1	1
153	I	5	1	M	A	NA	1	11.4	0.66	Prse_TLR4_Ht1.Prse_TLR4_Ht2	Prse_TLR7_Ht1	1	1	0	0	1	0	1	0
154	I	5	1	F	A	NA	1	11.4	0.66	Prse_TLR4_Ht1.Prse_TLR4_Ht2	Prse_TLR7_Ht1.Prse_TLR7_Ht2	1	1	0	0	1	1	1	1
155	I	5	1	M	A	NA	1	11.4	0.66	Prse_TLR4_Ht2.Prse_TLR4_Ht2	Prse_TLR7_Ht1	0	1	0	0	1	0	0	0
156	I	5	1	M	A	NA	1	11.4	0.66	Prse_TLR4_Ht1.Prse_TLR4_Ht2	Prse_TLR7_Ht1	1	1	0	0	1	0	1	0
157	I	5	1	M	A	6950	1	11.4	0.66	Prse_TLR4_Ht1.Prse_TLR4_Ht1	Prse_TLR7_Ht2	1	0	0	0	0	1	0	0
158	I	5	1	F	A	4000	1	11.4	0.66	Prse_TLR4_Ht2.Prse_TLR4_Ht2	Prse_TLR7_Ht1.Prse_TLR7_Ht1	0	1	0	0	1	0	0	0

Supplementary File 2: Detection of *Hepacivirus* infections

RNA from pools of blood from ten animals were extracted and screened for the presence of the *Hepacivirus* using a reactive nested reverse transcription-PCR as described in *Drexler* et al. (2013). The RNA of all individuals of positive pools were subsequently extracted (using 0.25 to 1 µl blood) and all individual samples were tested separately (n= 841). A 987 bp long fragment of the NS3 region of the protease/helicase peptide was targeted using a hemi-nested RT-PCR assay newly developed for the screening of the *Hepacivirus*. The primer pair used for the first PCR were HepaciPsem-F (5'-AGCCGCTGCTGATGAACAAGG-3') and HepaciPsem-R (5'-CRTTTGGRATGGTKGAGGCATC-3') and for the second PCR HepaciPsem-F and HepaciPsem-Rnest (5'-GAT GCC TTG KGC TGA DAR TTC YG-3').

The set up for the first PCR consisted of 600 nmol of forward and reverse primer each, 2x reaction buffer from the SuperScript III OneStep RT_PCR Kit (Life Technologies), 0.4 µl of a 50 mM magnesium sulfate solution (Life Technologies), 1 µg of PCR-grade BSA (Roche), 1 µL enzyme mix, and 5 µL RNA extract, summing up to a total volume of 25 µl. The conditions of the first PCR to perform the reverse transcription were set to 20 min at 55 °C, 3 min at 94 °C, followed by 10 cycles of 94 °C for 15 s, 60 °C (with a decrease of 1 °C per cycle) for 20 s and 72 °C for 60 s, followed by 2 min at 72 °C for final elongation.

The protocol for the second PCR consisted of an initial denaturation step of 3 min at 94 °C, followed by 45 cycles of 94 °C for 15 s, 56 °C for 20 s and 72 °C for 60 s and final elongation at 72 °C for 2 min. Reagents were mixed as following for the second PCR: 1 µl of the first PCR set up, 400 nmol of forward and reverse primer each, 10x Platinum Taq Buffer from Life Technologies, 200 nmol of deoxynucleotide triphosphates, 2.5 mmol of $MgCl_2$ and 1U of Platinum Taq polymerase from Life Technologies.

Samples were controlled for amplification success using gel-electrophoresis with ethidium bromide for staining and every second *Hepaci*-positive individual was Sanger-sequenced for further confirmation of viral infection. For further details see *Schmid* et al. 2018.

Supplementary File 3: Illustration of the three most common nematode egg morphotypes detected in *P. semispinosus* in the present study.

Nematode morphotype 1

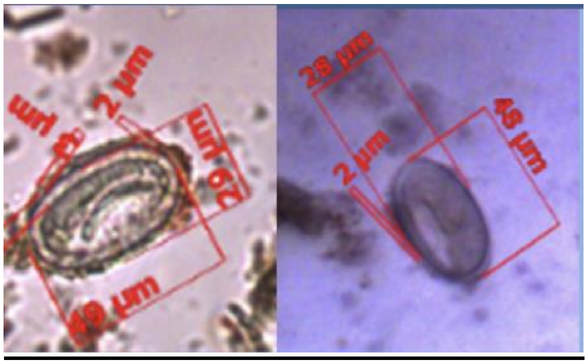

Nematode morphotype 3

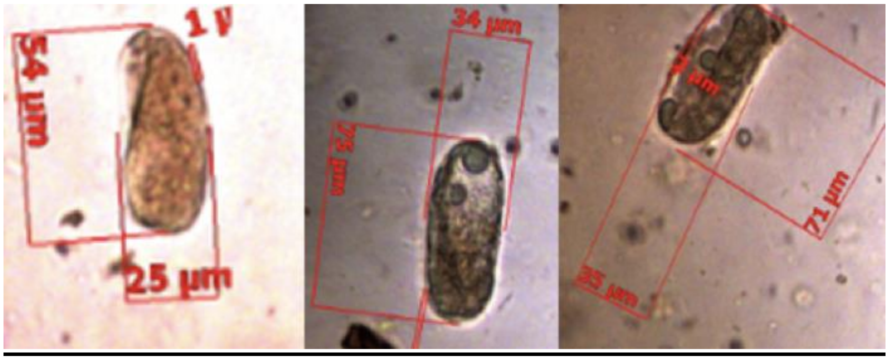

Nematode morphotype 6

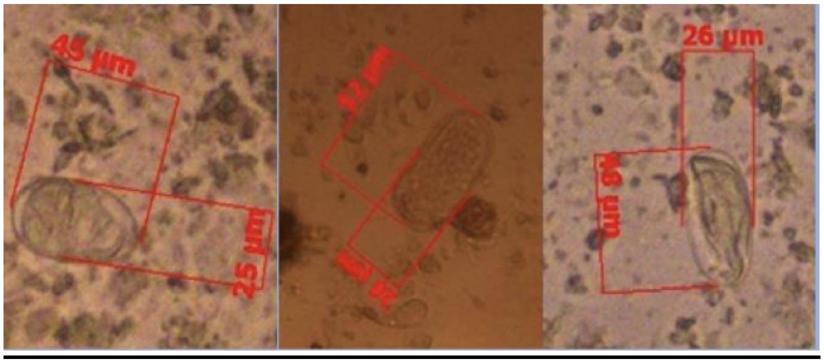

Supplement: Supplementary file 2 — Supplementary Files 1_3 [file 41437_2020_331_MOESM2_ESM.pdf]
