# Supplementary material for: Pathogen-associated selection on innate immunity genes (TLR4, TLR7) in a neotropical rodent in landscapes differing in anthropogenic disturbance
Source: Heredity (Edinb). 2020 Jul 2;125(4):184–99. doi: 10.1038/s41437-020-0331-y (PMC7490709; doi:10.1038/s41437-020-0331-y)
Supplement: Supplementary file 3 — Supplementary Figures 1_3 [file 41437_2020_331_MOESM3_ESM.pdf]

Supplementary Figure 1

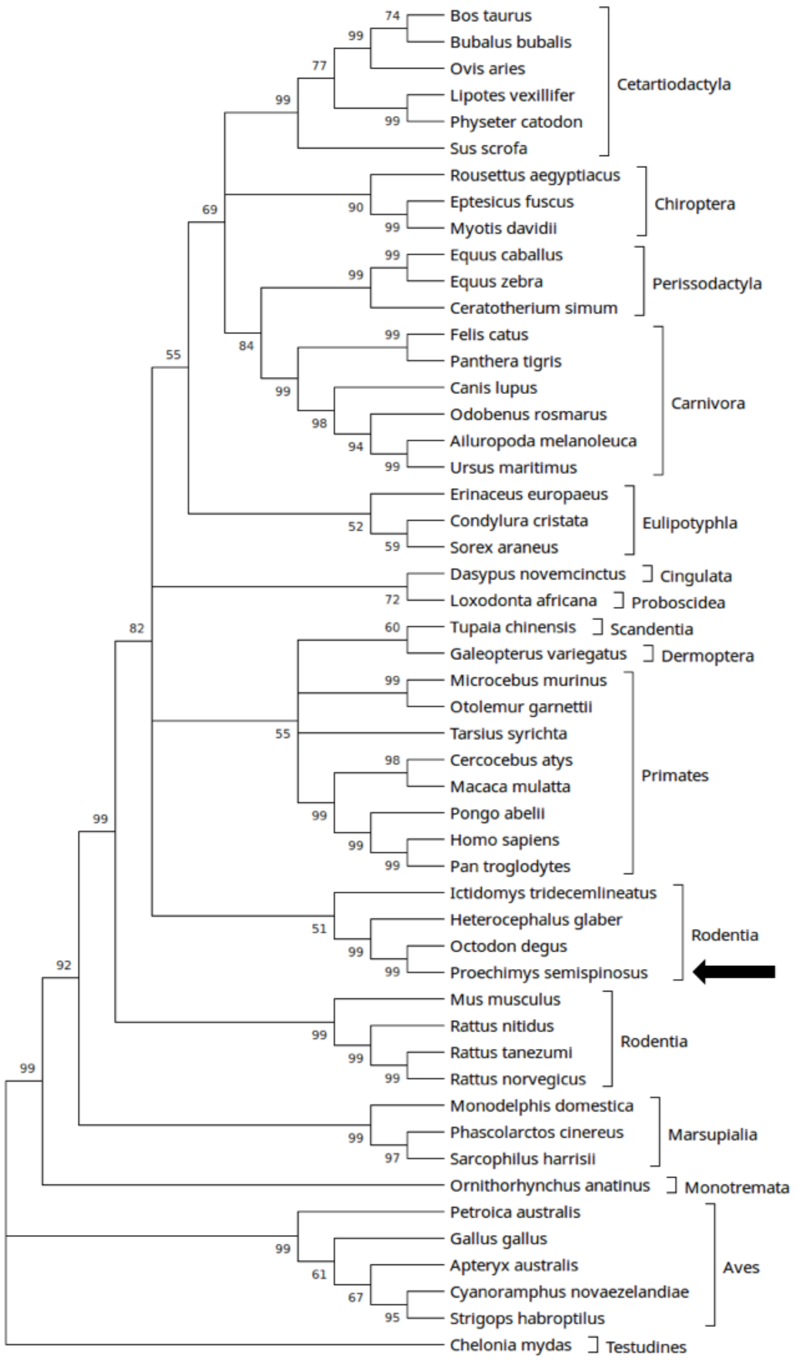

Supplementary Figure 1: Phylogenetic relationship of TLR4 sequences of mammals rooted with a reptile sequences. Accession numbers are summarized in Table S1. Bootstraps values > 50 are displayed. The arrow indicates the placement of our study species.

Supplementary Figure 2

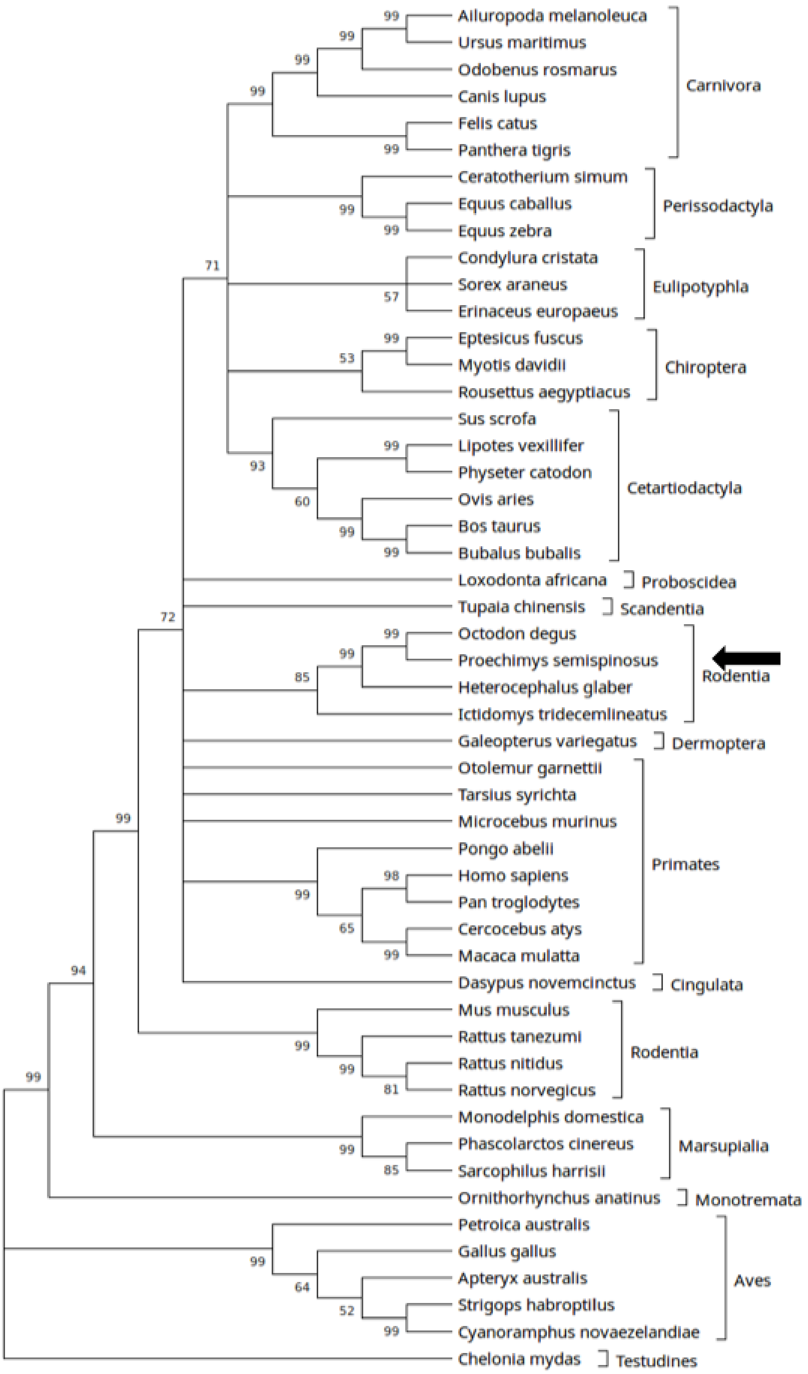

Supplementary Figure 2: Phylogenetic relationship of the TLR7 sequences of mammals rooted with a reptile sequence. Accession numbers are summarized in Table S1. Bootstraps values > 50 are displayed. The arrow indicates the placement of our study species.

Supplementary Figure 3

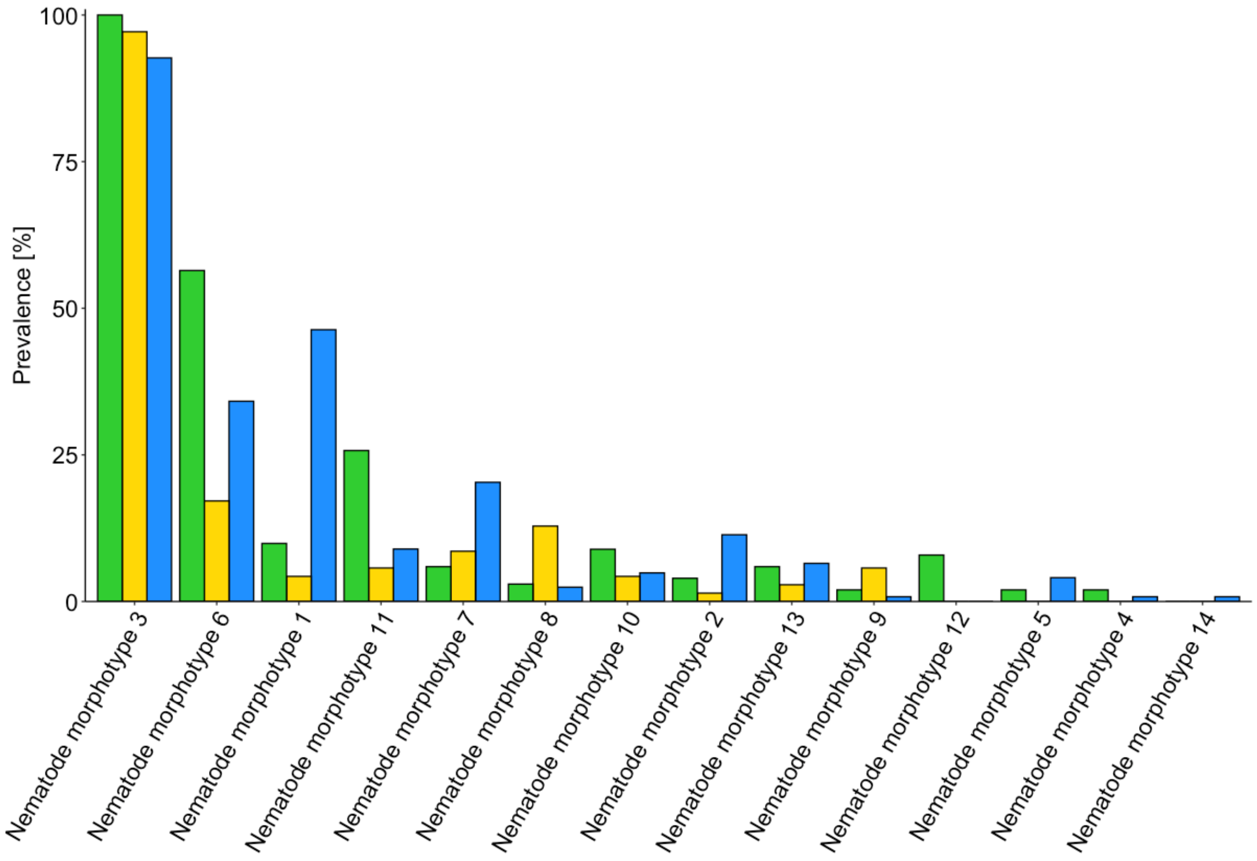

Supplementary Figure 3: Prevalence of nematode infections detected in *Proechimys semispinosus* across landscapes (C green, A yellow and I blue).
